# Adverse drug events associated with hydroxychloroquine: A real-world pharmacovigilance study from 2004 to 2024 using the FAERS database

**DOI:** 10.1097/MD.0000000000051285

**Published:** 2026-10-30

**Authors:** Shiwei Yuan, Zhen Li, Yongchang Yuan, Weifeng Lin, Yuan Zhang, Yongying Zhong, Jinyu Lin, Dong Chen, Wei Guo, Anning Zhu, Siyu Liu

**Affiliations:** a Longyan Traditional Chinese Medicine Hospital of Xiamen University, School of Medicine, Xiamen University, Longyan, Fujian, China; b Yueyang Hospital of Integrated Traditional Chinese and Western Medicine, Shanghai University of Traditional Chinese Medicine, Shanghai, China; c Fujian University of Traditional Chinese Medicine, Fuzhou, Fujian, China.

**Keywords:** FAERS, HCQ, pharmacovigilance, real world

## Abstract

**Background:**

Hydroxychloroquine (HCQ) is an antimalarial and immunomodulatory drug widely used to treat autoimmune diseases and other conditions. Despite its significant efficacy, HCQ can cause adverse effects such as gastrointestinal issues, retinal toxicity, and cardiotoxicity. As the application of HCQ in immunotherapy expands, its safety and long-term effects need to be evaluated through big data and clinical observations. In a post-marketing surveillance study conducted from the first quarter of 2004 to the third quarter of 2024, we analyzed HCQ-related adverse event (AE) from the The FDA’s Adverse Event Reporting System (FAERS) database, aiming to provide clinical references for its use.

**Methods:**

This retrospective pharmacovigilance study, based on the FAERS database, aimed to explore the association between HCQ and AEs. AE data from 2004 to 2024 were collected, with AE reports of the primary suspected drugs retrieved from the FAERS database. We filtered and analyzed reports related to HCQ use. Four different methods – reporting odds ratio, proportional reporting ratio, the multi-item gamma Poisson shrinker, and Bayesian confidence propagation neural network – were applied to perform disproportionality analysis on the AEs associated with HCQ.

**Results:**

The year 2020 had the highest number of AE reports, accounting for 20.44% of the total. In gender-based analysis, women were more likely to report AEs such as rheumatoid arthritis, disease exacerbation, drug intolerance, nausea, and pain, while men were more prone to report ECG QT prolongation and acute kidney injury. The study highlighted the differences in AE distribution across age groups and genders and pointed out that most AEs occurred within 1 month of starting HCQ; however, the risk of AEs remained even after 2 years, emphasizing the importance of long-term monitoring. The findings provided a reference for healthcare professionals and policymakers in developing safer drug usage guidelines.

**Conclusion:**

This study emphasizes that HCQ-related adverse reactions are influenced by factors such as gender, age, and underlying diseases, revealing the potential risks associated with the widespread use of HCQ, particularly the risks related to severe adverse reactions. It underscores the importance of continuous drug safety monitoring and suggests the need for individualized risk assessments in clinical settings, especially for patients on long-term use or combination therapies.

## 1. Introduction

Hydroxychloroquine (HCQ) is a synthetic derivative of chloroquine, with both antimalarial and immunomodulatory effects. As a weakly basic compound, it also exhibits antimalarial activity.^[1]^ In addition to its use in treating malaria, HCQ is widely employed for the treatment of autoimmune diseases, such as systemic lupus erythematosus (SLE) and rheumatoid arthritis (RA), through the inhibition of immune cell activation, particularly in regulating macrophages and T cells. During the process of immune modulation, HCQ interferes with antigen presentation, the release of inflammatory mediators, and the production of cytokines, thereby reducing the intensity of autoimmune responses, alleviating symptoms, and improving disease outcomes.^[1]^ In the treatment of SLE, HCQ effectively controls disease progression, alleviates joint pain, skin rashes, and other visceral damages, and slows the progression of the disease.^[2]^ For RA, HCQ exerts its anti-inflammatory effects to reduce joint swelling and pain, and it helps to slow the onset of joint damage.^[3]^ The immunomodulatory properties of HCQ also suggest its potential application in other autoimmune diseases, such as Sjögren’s syndrome and dermatomyositis. HCQ has been widely approved for the treatment of the aforementioned conditions, and dosage adjustments based on the patient’s specific condition are crucial to minimize side effects.^[4]^

Although HCQ has demonstrated favorable therapeutic effects, it may also cause adverse reactions. Common side effects include gastrointestinal symptoms, such as nausea, vomiting, and diarrhea, which are generally mild and tend to resolve with prolonged use.^[5]^ More serious side effects include retinal toxicity and cardiac toxicity. Long-term, high-dose use of HCQ may lead to retinal damage, manifested as blurred vision or color vision abnormalities, and in extreme cases, irreversible blindness. Therefore, regular ophthalmologic checkups are recommended, especially for long-term users. Cardiac toxicity, characterized by QT interval prolongation, may lead to fatal arrhythmias, particularly in patients with preexisting heart conditions or those taking other medications that affect the QT interval. Special caution is needed in these cases. Additionally, HCQ may cause skin reactions, such as rashes and photosensitivity. Therefore, it should be used cautiously in patients with a history of allergies.^[6]^ In clinical practice, adverse reactions to the drug should be managed through dose adjustments, supportive treatments (such as anti-nausea medications and ophthalmic examinations), and timely discontinuation of the drug when necessary.

In order to ensure the safety and efficacy of HCQ in clinical applications, especially during its widespread use in immunomodulatory treatments, drug safety monitoring is crucial. As the user population expands, the long-term safety of HCQ still requires continuous clinical observation and big data analysis for evaluation. Real-time monitoring of adverse drug reactions can be conducted through various methods, including clinical trials, adverse event (AE) data reported by patients, and regulatory agency reporting systems (such as the FDA’s Adverse Events Reporting System [FAERS]).^[7]^ The application of data mining technology enables the rapid identification of potential safety issues and undiscovered side effects after widespread use, thus providing more comprehensive treatment guidance for clinicians. Many researchers have delved into its mechanisms of action in an attempt to assess its potential therapeutic effects and side effects in different patient populations. Systematic drug safety monitoring conducted worldwide contributes to improving treatment protocols and enhancing the overall safety of drug use.^[8]^

Considering that HCQ is a versatile drug with significant efficacy in treating malaria and immune-related diseases, and the lack of evidence of HCQ AE in the real world, we conducted a post-marketing surveillance study to assess HCQ-related AE in patients using the drug from the first quarter of 2004 to the third quarter of 2024, based on FAERS data. We comprehensively analyzed the systemic-specific side effects of HCQ, their onset times, and gender-based differences. The findings of this study provide clinicians and health policymakers with some recommendations for monitoring drug adverse reactions and offer partial guidance for the safe clinical use of HCQ.

## 2. Methods

### 2.1. Study design and data sources

Data source: a retrospective pharmacovigilance study was conducted based on the FAERS database to investigate the correlation between HCQ and potential AEs. FAERS is a critical database of the U.S. Food and Drug Administration (FDA) for collecting AE information reported by patients and medical professionals during medication use.^[9]^ This study collected data from the FAERS database from the first quarter of 2004 to the third quarter of 2024. To ensure the specificity and accuracy of the study, the search was limited to the generic name “Hydroxychloroquine” and the brand name “Plaquenil” as the primary suspect (PS) drugs. Using MySQL software tools (SAS), data were collected, preprocessed, and cleaned to ensure accuracy and completeness. In accordance with the FDA-recommended method for removing duplicate reports, we selected the primary identifier (PRIMARYID), case identifier (CASEID), and FDA_DT fields from the DEMO table. We first sorted by CASEID, FDA_DT, and then by PRIMARYID. For reports with the same CASEID, we retained the report with the maximum FDA_DT value. For reports with the same CASEID and FDA_DT, we retained the report with the maximum PRIMARYID value.^[10,11]^ After deduplication, we deleted reports based on the CASEIDs listed in the deleted report list.^[12]^ Subsequently, the data were mapped to RxNorm and Medical Dictionary for regulatory activities (MedDRA) tools. Additionally, for preferred terms (PTs), standardization and translation were performed to ensure data consistency. To present the data, this study merged AE reports with the same PT. Meanwhile, using the System Organ Class (SOC) method, PTs were classified and organized to better summarize and analyze the characteristics of AEs. After data preprocessing, we obtained 18,289,374 DEMO reports, 66,422,789 DRUG cases, and 53,463,446 REAC records (Fig. 1).

**Figure 1.**
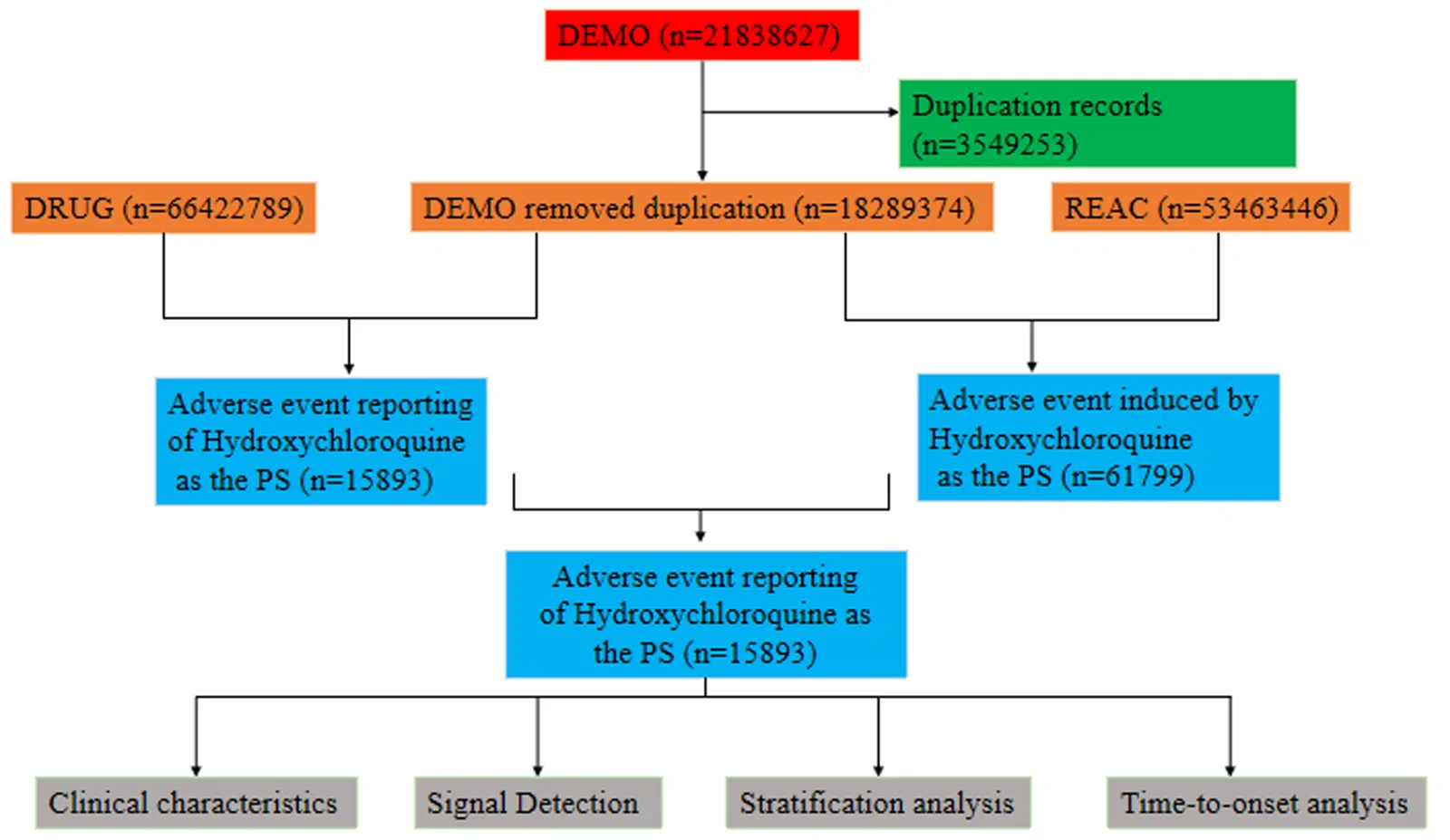
Flowchart. The process of mining HCQ data using the FAERS database. FAERS = The FDA’s Adverse Event Reporting System, HCQ = hydroxychloroquine.

### 2.2. Statistical analysis

Drugs in FAERS reports are categorized into 4 modes: PS, SS (secondary suspect), C (concomitant), and I (interaction). To improve accuracy, only drugs with HCQ as PS in the AE action code were retained, thus screening 15,893 AE reports (HCQ-related AEs) for HCQ administration.^[13]^ MedDRA is a standardized medical terminology that facilitates the recording and reporting of AE data globally.^[14,15]^ Its hierarchy includes multiple levels, from lower-level terms to SOC.^[16]^ SOC is the highest-level term in MedDRA used for classifying and reporting AE in drug safety monitoring and reporting systems. To summarize and analyze AE characteristics in a structured manner, all AEs collected were encoded using PTs and then mapped to the corresponding highest SOC level in MedDRA (Version 26.0).^[17,18]^ A total of 61,799 PTs induced by HCQ as PS (HCQ-related PTs) were identified. In pharmacovigilance studies, disproportionality analysis is a tool method used to identify and detect drug-related AE signals. To improve the reliability of the results, we adopted multiple disproportionality analysis methods, among which the reporting odds ratio (ROR) has shown good performance in early signal detection. ROR and proportional reporting ratio (PRR) methods are widely used to detect drug-related AE signals.^[19]^

The core advantage of ROR lies in directly reflecting the association strength between the target drug and the target AE. Its calculation logic can intuitively quantify the difference between the “probability of a certain AE occurring after using HCQ” and the “probability of the same AE occurring without using HCQ.” The results are easy to explain and conform to the cognitive habits of clinical medical staff and policymakers. This is highly consistent with the core objective of this study, which is to provide clear references for clinical medication. Meanwhile, ROR demonstrates stable signal detection performance in large sample size data, without relying on complex distribution assumptions, and can effectively avoid the bias that may occur in small sample algorithms.

There are also Bayesian confidence propagation neural network (BCPNN) and multi-item gamma-Poisson shrinker (MGPS).^[20]^ In cases with fewer drug AE reports, MGPS and BCPNN methods still have strong signal detection capabilities.^[21,22]^ We selected ROR as the main signal mining method and used the disproportionality measurement method in a 2 × 2 contingency table to report AE data of HCQ and other drugs (Table 1). Subsequently, 4 statistical methods – ROR, PRR, BCPNN, and MGPS – were used in combination with all 4 thresholds to screen eligible positive signals (Table 2). Furthermore, to identify disproportional signals between males and females after HCQ administration, we adopted the formula of the ROR method. The ROR used here does not strictly adhere to the pharmacoepidemiological definition of ROR. Based on the 2 × 2 contingency table, we calculated the *P* value using the chi-square (χ2) test. Using the R software package “ggplot2” (Version 4.3.0),^[19]^ we created a volcano plot displaying the log2-transformed ROR values on the horizontal axis and the –log10-transformed corrected *P* values (*P*.adj, adjusted by FDR) on the vertical axis. When ROR > 1 and *P*.adj > .05, it indicates that female patients are more likely to report specific AEs than male patients. Conversely, when ROR < 1 and *P*.adj < .05, it suggests that male patients are more likely to report specific AEs than female patients.

**Table 1:** Four-grid table of disproportionality analysis method.

Item	Target number of adverse reactions reported	Number of other adverse reactions reported	Summation
Target drug	*a*	*b*	*a* + *b*
Other drugs	*c*	*d*	*c* + *d*
*Total*	*a* + *c*	*b* + *d*	N = *a* + *b* + *c* + *d*

AEs = adverse events; *a*: number of reports containing both the target drug and target adverse drug reaction, *b* = number of reports containing other adverse drug reaction of the target drug, *c* = number of reports containing the target adverse drug reaction of other drugs, *d* = number of reports containing other drugs and other adverse drug reactions.

**Table 2:** Principle of disproportionality measure and standard of signal detection.

Algorithms	Calculation formula	Threshold
ROR	$\begin{array}{cc} ROR=\frac{\left(a/c\right)}{\left(b/d\right)}=\frac{ad}{bc}\\ & SE(InROR)=\sqrt{\left(\frac{1}{a}+\frac{1}{b}+\frac{1}{c}+\frac{1}{d}\right)}\\ & 95\%CI=eIn(ROR)\pm 1.96\sqrt{\left(\frac{1}{a}+\frac{1}{b}+\frac{1}{c}+\frac{1}{d}\right)} \end{array}$	*a* ≥ 3 with a lower 95% CI > 1
PRR	$\begin{array}{cc} PRR=\frac{a/\left(a+b\right)}{c/(c+d)}\\ & SE(InPRR)=\sqrt{\frac{1}{a}-\frac{1}{a+b}+\frac{1}{c}-\frac{1}{c+d}}\\ & 95\%CI=eIn(PRR)\pm 1.96\sqrt{\left(\frac{1}{a}-\frac{1}{a+b}+\frac{1}{c}-\frac{1}{c+d}\right)} \end{array}$	*a* ≥ 3 with a lower 95% CI > 1
BCPNN	$IC=log2\;\frac{p(x,y)}{p(x)\;p(y)}=log2\frac{a(a+b+c+d)}{\left(a+b)(a+c\right)}$ $E(IC)=log2\frac{(a+\gamma 11)(a+b+c+d+\alpha )(a+b+c+d+\beta )}{(a+b+c+d+\gamma )(a+b+\alpha 1)(a+b+\beta 1)}$ $V(IC)=\frac{1}{{\left(In2\right)}^{2}}\{\left[\frac{(a+b+c+d)\text{\textasciitilde{}}\text{-}a+\gamma -\gamma 11}{(a+\gamma 11)(1+a+b+c+d+\gamma )}\right]$$+\left[\frac{\left(a+b+c+d)\text{\textasciitilde{}}\text{-}(a+b\right)+\alpha -\alpha 1}{\left(a+b+c+d)\text{\textasciitilde{}}\text{-}(a+b\right)+\alpha -\alpha 1}\right]+\left[\frac{\left(a+b+c+d)\text{\textasciitilde{}}\text{-}(a+c\right)+\beta -\beta 1}{(a+c+\beta 1)(1+a+b+c+d+\beta )}\right]\}$ $\gamma =\gamma 11\frac{(a+b+c+d+\alpha )(a+b+c+d+\beta )}{(a+b+\alpha 1)(a+c+\beta 1)}$ $IC-2SD=E(IC)-2\sqrt{v(IC)}$ α1=β1 = 1;α=β = 2;γ11 = 1	(−): E(IC) ≤ 0 (+):0 < E(IC) ≤ 1.5: (++):1.5 < E(IC) ≤ 3: (+++):E(IC) > 3
MGPS	$\begin{array}{cc} EBGM=a(a+b+c+d)/((a+c)(a+b))\\ & EBGM05={e^{in}}^{(EBGM)-1.64(1/a+1/b+1/c+1/d\hat{)}0.5} \end{array}$	EBGM05 > 2,*a* > 0

95% CI = 95% confidence interval, *a* = number of reports containing both the target drug and target adverse drug reaction, *b* = number of reports containing other adverse drug reaction of the target drug, BCPNN = Bayesian confidence propagation neural network, *c* = number of reports containing the target adverse drug reaction of other drugs, *d* = number of reports containing other drugs and other adverse drug reactions, E(IC) = the IC expectations, EBGM = empirical Bayesian geometric mean, EBGM05 = the lower limit of 95% CI of EBGM, IC = information component, IC025 = the lower limit of 95% CI of the IC, MGPS = the multi-item gamma Poisson shrinker, N = the number of reports, PRR = proportional reporting ratio, ROR = reporting odds ratio, V(IC) = the variance of IC, χ^2^ = chi-squared.

## 3. Results

### 3.1. Basic information on AE reports

This study extracted data from the FAERS database from the first quarter of 2004 to the third quarter of 2024, totaling 21,838,627 AE reports. After data cleaning and drug screening, 15,839 HCQ-related AE reports were included in the analysis. As observed in Table 3, males accounted for the largest proportion of HCQ AE reports at 55.2% (8772 cases), followed by females at 23.7% (3766 cases), with 21.1% (3355 cases) lacking gender information. Regarding age data, 36.9% (5865 cases) had no detailed age statistics. Among the available data, the 18 to 64.9 age group had the highest number of AEs at 42.5% (6748 cases), followed by 65 to 85 years (16.5%, 2615 cases), under 18 years (3.3%, 521 cases), and over 85 years (0.9%, 144 cases). In terms of reporters, pharmacists were the primary group (34.5%, 5488 cases), followed by physicians (22.9%, 3642 cases) and consumers (17.9%, 2847 cases). The vast majority of AEs (98.76%) originated from the U.S., followed by Canada (15.3%, 2431 cases) and France (8.5%, 1356 cases). Excluding data from the fourth quarter of 2024, the highest number of AE reports (20.44% of the total) occurred in 2020, as shown in the annual distribution in Figure 2. In clinical outcomes, among severe AEs (excluding unspecified cases), there were 999 deaths, with a mortality rate of 6.3%, indicating serious drug-related AEs. Reports of first-time or prolonged hospitalization accounted for 18.8%, further highlighting the drug’s potential severity.

**Table 3:** Basic information table.

Basic	HCQ	Overall FAERS
Sex		
F	8772 (55.2%)	9,690,695 (53.24%)
M	3766 (23.7%)	6,360,074 (34.94%)
Missing	3355 (21.1%)	2,150,177 (11.81%)
WT		
<50 kg	285 (1.8%)	396,937 (2.18%)
>100 kg	157 (1.0%)	480,540 (2.64%)
50–100 kg	1243 (7.8%)	2,824,385 (15.52%)
Missing	14,208 (89.4%)	14,499,084 (79.66%)
Age		
<18 yr	521 (3.3%)	782,641 (4.3%)
>85 yr	144 (0.9%)	218,411 (1.2%)
18–64.9 yr	6748 (42.5%)	10,829,563 (59.5%)
65–85 yr	2615 (16.5%)	2,420,726 (13.3%)
Missing	5865 (36.9%)	3,949,605 (21.7%)
OCCP_COD		
CN	2847 (17.9%)	8,255,515 (45.36%)
HP	5488 (34.5%)	1,447,045 (7.95%)
LW	3 (0.0%)	521,470 (2.87%)
MD	3642 (22.9%)	4,060,743 (22.31%)
OT	2828 (17.8%)	1,970,033 (10.82%)
PH	485 (3.1%)	1,168,963 (6.42%)
RN	4 (0.0%)	8341 (0.05%)
Missing	596 (3.8%)	768,826 (4.22%)
OUTC_COD		
CA	71 (0.4%)	43,277 (0.24%)
DE	999 (6.3%)	1,667,819 (9.16%)
DS	336 (2.1%)	223,323 (1.23%)
HO	2980 (18.8%)	3,263,686 (17.93%)
LT	615 (3.9%)	418,760 (2.30%)
OT	8618 (54.2%)	4,677,257 (25.70%)
RI	25 (0.2%)	37,782 (0.21%)
Missing	2249 (14.2%)	7,869,042 (43.23%)
REPORTER_ COUNTRY		
United States	5881 (37.0%)	12,318,567 (67.68%)
Canada	2431 (15.3%)	625,721 (3.44%)
France	1356 (8.5%)	520,201 (2.86%)
Spain	834 (5.2%)	147,381 (0.81%)
Italy	799 (5.0%)	243,335 (1.34%)
Missing	2 (0.0%)	677,426 (3.72%)

CN = clinical nurse, FAERS = The FDA’s Adverse Event Reporting System, HCQ = hydroxychloroquine, HP = health professional, LW = laborer/worker, MD = medical doctor, OCCP_COD = occupational code, OT = occupational therapist, PH = public health worker, RN = registered nurse.

**Figure 2.**
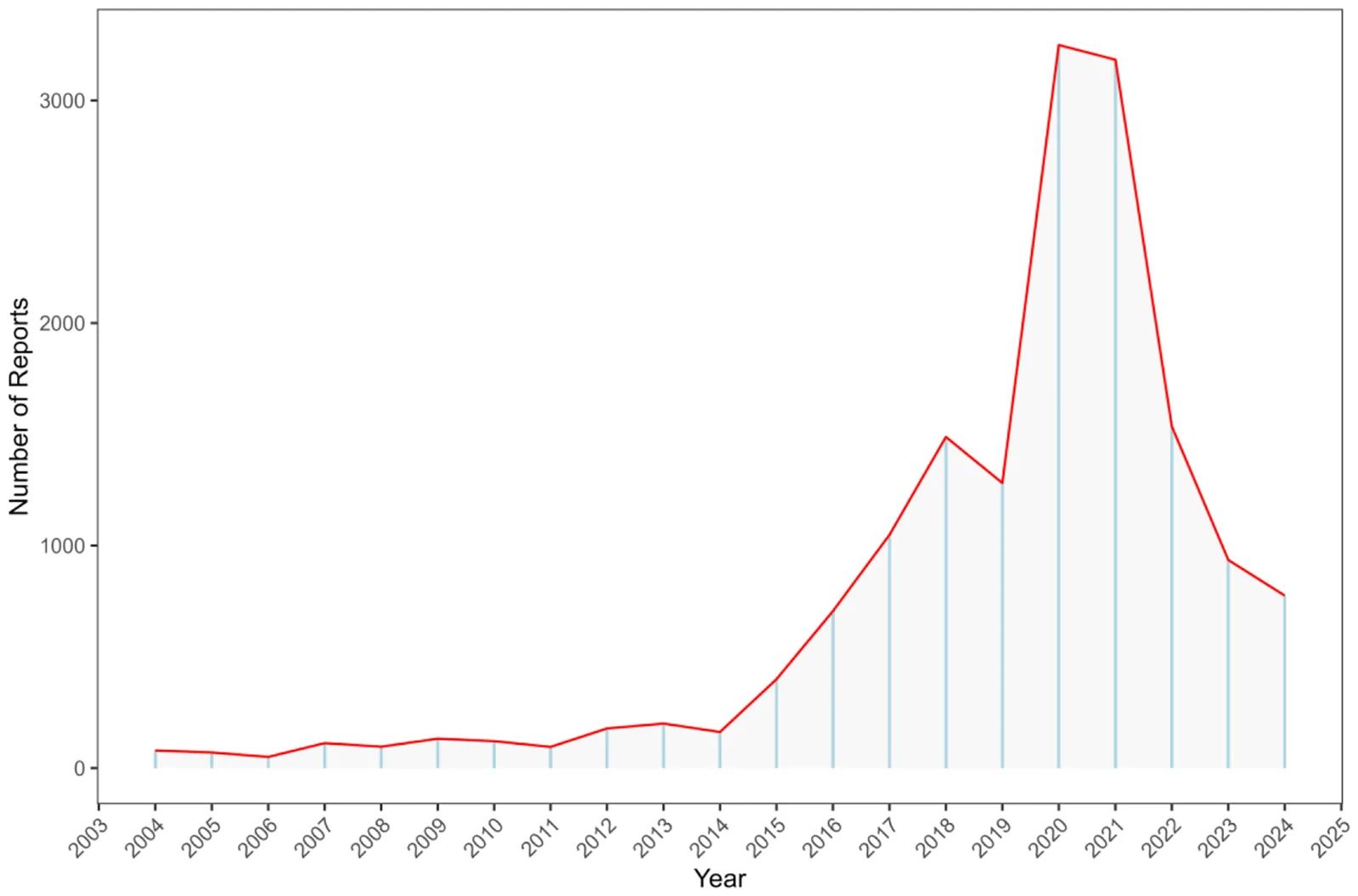
2004Q1 to 2024Q3 annual report. Display the trend chart of the number of HCQ adverse reactions recorded in the FAERS database from 2004Q1 to 2024Q3. FAERS = The FDA’s Adverse Event Reporting System, HCQ = hydroxychloroquine.

### 3.2. Signal detection

#### 3.2.1. Signal detection statistics at the SOC level

Based on 4 calculation methods, this study identified 27 HCQ-related AE signals in SOC categories, as shown in Figure 3. Using the strictest ROR method for ranking, the 3 most prominent categories were: pregnancy, puerperium, and perinatal conditions (n = 902, ROR 3.41), eye disorders (n = 2555, ROR 2.09), and musculoskeletal and connective tissue disorders (n = 6312, ROR 2.02). According to the number of reports, the top 3 SOC categories were general disorders and administration site conditions, musculoskeletal and connective tissue disorders, and injury, poisoning, and procedural complications (Fig. 4).

**Figure 3.**
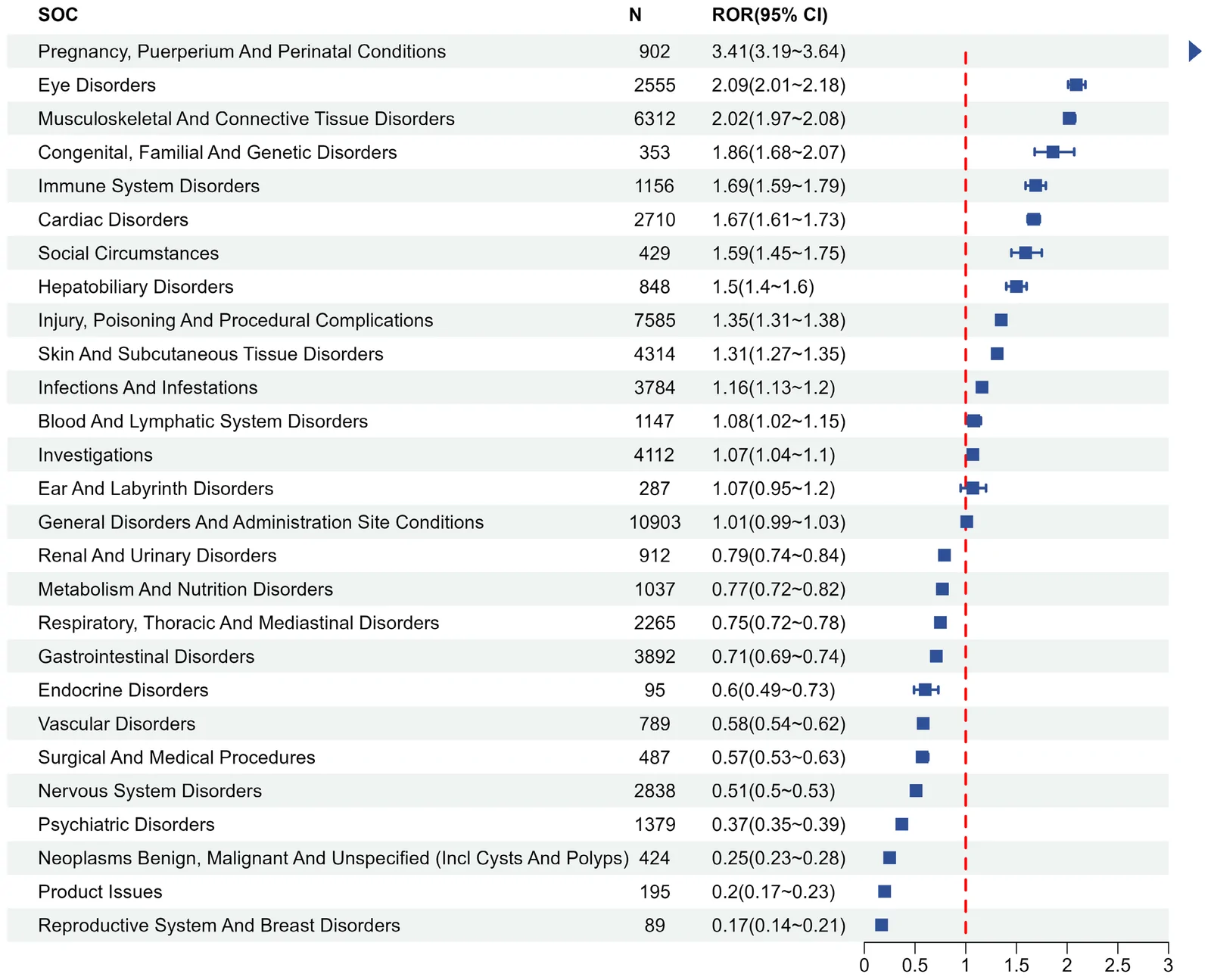
Lists the SOCs according to the top 27 confidence levels in the ROR. List the confidence forest chart of 27 SOCs in HCQ based on the strength of ROR confidence. CI = confidence interval, HCQ = hydroxychloroquine, ROR = reporting odds ratio, SOC = System Organ Class.

**Figure 4.**
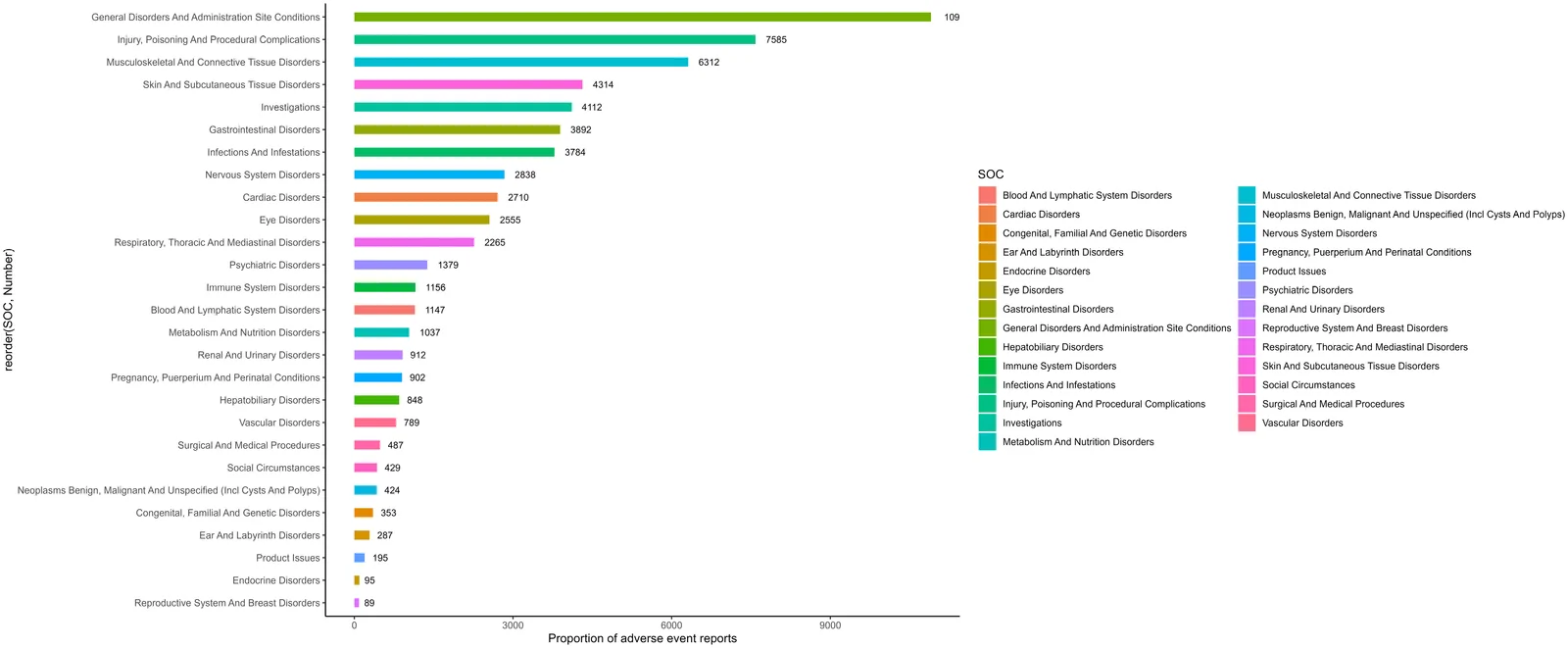
SOC ranking based on number of reports. The distribution of SOCs to which HCQ adverse reaction PT belongs, with the number of cases on the horizontal axis and the names of SOCs on the vertical axis. HCQ = hydroxychloroquine, PT = preferred term, SOC = System Organ Class.

#### 3.2.2. Signal detection at the PT level

After HCQ administration, we sorted the top 50 PTs by ROR (95% confidence interval [CI]) values and marked their corresponding SOC categories (Fig. 5). The top 3 positive PTs were chondrodysplasia punctata, red blood cell vacuolization, and T-cell receptor gene rearrangement test. In terms of occurrence frequency, the top 3 PTs (Fig. 6) were RA, condition aggravated, and drug intolerance. Except for RA, the top 5 PTs had ROR values < 10. PTs meeting positive criteria across all 4 algorithms (ROR, PRR, BCPNN, and MGPS) were summarized by SOC (Table 4), visualized in Figure 7, and cross-validated in Figure 8.

**Table 4:** Summary of preferred terms (PT) and system organ classes (SOC) that are positive according to all 4 algorithms: ROR, PRR, BCPNN, and MGPS.

SOC	PT	a	ROR	IC025	PRR	EBGM05
Blood and Lymphatic System Disorders	Leukopenia	98	1.91 (1.57–2.33)	0.93 (0.64)	1.91 (42.61)	1.91 (1.62)
	Eosinophilia	79	4.50 (3.61–5.62)	2.16 (1.84)	4.5 0(213.91)	4.48 (3.72)
	Lymphadenopathy	50	1.36 (1.03–1.80)	0.45 (0.04)	1.36 (4.83)	1.36 (1.08)
	Lymphopenia	49	3.42 (2.58–4.53)	1.77 (1.36)	3.42 (83.48)	3.41 (2.69)
	Hemolytic Anemia	47	5.06 (3.80–6.74)	2.33 (1.91)	5.06 (152.13)	5.03 (3.96)
Cardiac Disorders	Cardiomyopathy	225	14.67 (12.86–16.74)	3.85 (3.65)	14.62 (2808.57)	14.4 (12.89)
	Cardiac Failure	136	1.64 (1.38–1.94)	0.71 (0.46)	1.63 (33.51)	1.63 (1.42)
	Cardiotoxicity	136	16.09 (13.58–19.07)	3.98 (3.73)	16.06 (1885.66)	15.78 (13.69)
	Cardiac Arrest	128	1.48 (1.24–1.76)	0.56 (0.31)	1.48 (19.6)	1.47 (1.28)
	Ventricular Tachycardia	116	6.79 (5.65–8.15)	2.75 (2.48)	6.78 (566.88)	6.73 (5.78)
Congenital, Familial and Genetic Disorders	Hypertrophic Cardiomyopathy	34	22.16 (15.77–31.15)	4.43 (3.94)	22.15 (669.38)	21.62 (16.26)
	Fabry’s Disease	14	97.11 (55.88–168.76)	6.45 (5.66)	97.09 (1196.3)	87.34 (55)
	Anterior Chamber Cleavage Syndrome	10	204.77 (102.74–408.14)	7.37 (6.42)	204.74 (1637.56)	165.56 (92.97)
	Lipidosis	9	175.92 (85.88–360.36)	7.19 (6.20)	175.89 (1299.3)	146.19 (80.23)
	Neonatal Lupus Erythematosus	8	245.72 (111.99–539.14)	7.58 (6.51)	245.69 (1516.31)	191.31 (99.13)
Ear and Labyrinth Disorders	Tinnitus	74	1.56 (1.24–1.96)	0.64 (0.30)	1.56 (14.69)	1.56 (1.28)
	Deafness	47	1.77 (1.33–2.36)	0.82 (0.41)	1.77 (15.78)	1.77 (1.39)
	Deafness Bilateral	18	10.66 (6.70–16.97)	3.4 (2.73)	10.66 (155.64)	10.54 (7.14)
	Deafness Neurosensory	17	5.25 (3.26–8.46)	2.39 (1.70)	5.25 (58.15)	5.23 (3.51)
	Deafness Unilateral	17	3.03 (1.88–4.87)	1.59 (0.91)	3.03 (22.99)	3.02 (2.03)
Endocrine Disorders	Cushingoid	14	4.4 (2.6–7.44)	2.13 (1.39)	4.4 (36.62)	4.38 (2.83)
	Cushing’s Syndrome	9	2.15 (1.12–4.14)	1.1 (0.19)	2.15 (5.54)	2.15 (1.24)
	Hyperparathyroidism Primary	3	8.3 (2.66–25.86)	3.04 (1.59)	8.29 (19.06)	8.23 (3.18)
Eye Disorders	Retinopathy	257	70.87 (62.39–80.51)	6.03 (5.84)	70.58 (16,293.33)	65.31 (58.70)
	Visual Impairment	206	1.68 (1.47–1.93)	0.75 (0.54)	1.68 (56.46)	1.68 (1.50)
	Maculopathy	187	52.19 (45.02–60.51)	5.62 (5.40)	52.04 (8827.41)	49.13 (43.41)
	Retinal Toxicity	177	222.51 (188.58–262.53)	7.46 (7.23)	221.87 (30,936.58)	176.57 (153.75)
	Visual Field Defect	121	16.07 (13.42–19.24)	3.98 (3.71)	16.04 (1675.02)	15.76 (13.56)
Gastrointestinal Disorders	Abdominal Discomfort	350	2.07 (1.86–2.30)	1.04 (0.89)	2.06 (191.39)	2.06 (1.89)
	Gastrointestinal Disorder	180	2.16 (1.86–2.50)	1.11 (0.89)	2.16 (111.37)	2.15 (1.90)
	Glossodynia	90	4.11 (3.34–5.05)	2.03 (1.73)	4.1 (210.37)	4.09 (3.44)
	Mouth Ulceration	45	2.12 (1.58–2.84)	1.08 (0.66)	2.12 (26.52)	2.12 (1.66)
	Coeliac Disease	30	3.06 (2.14–4.38)	1.61 (1.09)	3.06 (41.51)	3.05 (2.26)
General Disorders and Administration Site Conditions	Condition Aggravated	888	3.03 (2.84–3.24)	1.58 (1.49)	3.0(1189.45)	3.0 (2.84)
	Drug Intolerance	716	7.50 (6.97–8.08)	2.88 (2.77)	7.43 (3955.41)	7.37 (6.93)
	Treatment Failure	497	6.23 (5.7–6.8)	2.62 (2.49)	6.18 (2147.37)	6.15 (5.71)
	Drug Interaction	288	1.75 (1.56–1.96)	0.80 (0.63)	1.74 (91.37)	1.74 (1.58)
	Peripheral Swelling	283	1.83 (1.63–2.05)	0.87 (0.69)	1.82 (105.32)	1.82 (1.65)
Hepatobiliary Disorders	Hepatitis	157	6.05 (5.17–7.08)	2.59 (2.35)	6.04 (655.5)	6.0(5.26)
	Drug-Induced Liver Injury	76	2.71 (2.17–3.40)	1.44 (1.11)	2.71 (81.85)	2.71 (2.24)
	Hepatotoxicity	76	3.42 (2.73–4.29)	1.77 (1.44)	3.42 (129.74)	3.41 (2.82)
	Liver Disorder	66	1.46 (1.15–1.86)	0.55 (0.2)	1.46 (9.68)	1.46 (1.2)
	Liver Injury	66	3.07 (2.41–3.91)	1.61 (1.26)	3.07 (91.61)	3.06 (2.5)
Immune System Disorders	Drug Hypersensitivity	515	2.53 (2.32–2.76)	1.33 (1.2)	2.52 (470.45)	2.51 (2.34)
	Hypersensitivity	329	1.73 (1.55–1.93)	0.79 (0.63)	1.73 (100.88)	1.73 (1.58)
	Anaphylactic Reaction	72	1.34 (1.06–1.68)	0.42 (0.08)	1.34 (6.09)	1.34 (1.10)
	Hemophagocytic Lymphohistiocytosis	35	5.91 (4.24–8.24)	2.55 (2.07)	5.91 (141.68)	5.87 (4.45)
	Immunodeficiency	27	1.96 (1.35–2.87)	0.97 (0.43)	1.96 (12.75)	1.96 (1.43)
Infections and Infestations	Infection	184	1.28 (1.11–1.48)	0.35 (0.14)	1.28 (11.03)	1.28 (1.13)
	Herpes Zoster	116	1.94 (1.62–2.33)	0.96 (0.69)	1.94 (52.9)	1.94 (1.67)
	Septic Shock	85	1.96 (1.59–2.43)	0.97 (0.66)	1.96 (40.03)	1.96 (1.64)
	Lower Respiratory Tract Infection	59	1.35 (1.05–1.75)	0.43 (0.06)	1.35 (5.39)	1.35 (1.09)
	Staphylococcal Infection	49	1.40 (1.06–1.85)	0.49 (0.08)	1.40 (5.61)	1.40 (1.11)
Injury, Poisoning and Procedural Complications	Toxicity To Various Agents	506	3.05 (2.79–3.32)	1.60 (1.47)	3.03 (687.21)	3.02 (2.81)
	Intentional Overdose	146	2.32 (1.97–2.73)	1.21 (0.97)	2.32 (109.1)	2.31 (2.02)
	Infusion Related Reaction	85	1.32 (1.06–1.63)	0.39 (0.08)	1.31 (6.4)	1.31 (1.10)
	Wound	62	2.30 (1.79–2.95)	1.20 (0.83)	2.30 (45.22)	2.29 (1.86)
	Retinal Injury	27	34.77 (23.66–51.09)	5.06 (4.51)	34.76 (850.83)	33.45 (24.24)
Investigations	Electrocardiogram Qt Prolonged	579	16.19 (14.91–17.59)	3.98 (3.86)	16.05 (8027.54)	15.78 (14.72)
	Hepatic Enzyme Increased	198	2.93 (2.55–3.37)	1.55 (1.34)	2.93 (250.37)	2.92 (2.6)
	C-Reactive Protein Increased	157	4.28 (3.66–5.01)	2.09 (1.86)	4.27 (391.99)	4.26 (3.73)
	Red Blood Cell Sedimentation Rate Increased	118	10.42 (8.69–12.5)	3.36 (3.1)	10.40 (990.95)	10.29 (8.84)
	C-Reactive Protein Abnormal	118	21.13 (17.6–25.37)	4.37 (4.1)	21.1 (2204.98)	20.61 (17.69)
Metabolism and Nutrition Disorders	Hypokalemia	199	4.26 (3.71–4.90)	2.08 (1.88)	4.25 (492.97)	4.24 (3.77)
	Hypoglycemia	104	2.05 (1.69–2.49)	1.03 (0.75)	2.05 (55.80)	2.05 (1.74)
	Phospholipidosis	33	525.78 (340.95–810.81)	8.35 (7.77)	525.50 (10,722.8)	326.55 (227.28)
	Hypocalcemia	31	1.62 (1.14–2.30)	0.69 (0.18)	1.62 (7.26)	1.61 (1.20)
	Hypercalcemia	21	1.65 (1.08–2.54)	0.72 (0.11)	1.65 (5.40)	1.65 (1.15)
Musculoskeletal and Connective Tissue Disorders	Rheumatoid Arthritis	1247	11.16 (10.55–11.81)	3.44 (3.35)	10.95 (11,157.71)	10.83 (10.33)
	Arthralgia	696	1.66 (1.54–1.79)	0.72 (0.61)	1.65 (180.69)	1.65 (1.55)
	Joint Swelling	517	4.27 (3.91–4.66)	2.08 (1.95)	4.24 (1276.8)	4.23 (3.93)
	Synovitis	281	16.24 (14.42–18.27)	3.99 (3.82)	16.17 (3925.41)	15.89 (14.39)
	Systemic Lupus Erythematosus	267	8.29 (7.35–9.36)	3.03 (2.86)	8.26 (1688.74)	8.19 (7.4)
Neoplasms Benign, Malignant and Unspecified (Incl Cysts and Polyps)	Myelodysplastic Syndrome	29	1.91 (1.33–2.75)	0.93 (0.4)	1.91 (12.52)	1.91 (1.41)
	Meningioma	13	3.88 (2.25–6.69)	1.95 (1.18)	3.88 (27.67)	3.87 (2.45)
	Non-Hodgkin’s Lymphoma	12	1.90 (1.08–3.35)	0.93 (0.12)	1.90 (5.12)	1.90 (1.18)
	Breast Cancer Stage Iii	11	1.86 (1.03–3.36)	0.90(0.06)	1.86 (4.38)	1.86 (1.13)
	Lymphoproliferative Disorder	7	2.37 (1.13–4.98)	1.24 (0.22)	2.37 (5.53)	2.37 (1.27)
Nervous System Disorders	Sciatica	37	2.38 (1.72–3.29)	1.25 (0.78)	2.38 (29.54)	2.38 (1.81)
	Posterior Reversible Encephalopathy Syndrome	35	3.79 (2.72–5.29)	1.92 (1.44)	3.79 (71.58)	3.78 (2.86)
	Myasthenia Gravis	32	4.59 (3.24–6.50)	2.19 (1.69)	4.59 (89.34)	4.57 (3.42)
	Neuromyopathy	32	29.7 (20.88–42.25)	4.84 (4.33)	29.68 (857.39)	28.73 (21.39)
	Fine Motor Skill Dysfunction	16	5.63 (3.44–9.21)	2.49 (1.78)	5.63 (60.52)	5.60 (3.71)
Pregnancy, Puerperium and Perinatal Conditions	Premature Delivery	123	7.36 (6.16–8.79)	2.87 (2.61)	7.34 (668.54)	7.29 (6.28)
	Low Birth Weight Baby	111	13.69 (11.35–16.52)	3.75 (3.48)	13.67 (1283.15)	13.47 (11.51)
	Abortion Spontaneous	65	1.54 (1.21–1.96)	0.62 (0.27)	1.54 (12.24)	1.54 (1.25)
	Live Birth	54	6.99 (5.35–9.13)	2.79 (2.40)	6.98 (274.61)	6.93 (5.54)
	Foetal Growth Restriction	50	8.3 (6.28–10.97)	3.04 (2.63)	8.3 (317.77)	8.23 (6.52)
Product Issues	Product Substitution Issue	100	1.68 (1.38–2.05)	0.75 (0.46)	1.68 (27.58)	1.68 (1.43)
Psychiatric Disorders	Suicide Attempt	84	1.33 (1.08–1.65)	0.41 (0.10)	1.33 (6.98)	1.33 (1.11)
	Mental Status Changes	52	1.8 (1.37–2.36)	0.85 (0.45)	1.80 (18.41)	1.80 (1.43)
	Delirium	47	1.35 (1.01–1.80)	0.43 (0.02)	1.35 (4.28)	1.35 (1.06)
	Sleep Disorder Due To General Medical Condition, Insomnia Type	22	3.95 (2.6–6.0)	1.98 (1.37)	3.95 (48.17)	3.93 (2.77)
	Fear Of Injection	13	2.04 (1.18–3.51)	1.03 (0.25)	2.04 (6.86)	2.04 (1.29)
Renal and Urinary Disorders	Acute Kidney Injury	182	1.19 (1.03–1.38)	0.25 (0.04)	1.19 (5.67)	1.19 (1.06)
	Proteinuria	54	2.88 (2.2–3.76)	1.52 (1.13)	2.88 (66)	2.87 (2.30)
	Lupus Nephritis	50	31.94 (24.09–42.37)	4.95 (4.53)	31.92 (1443.98)	30.81 (24.33)
	Renal Phospholipidosis	42	737.57 (488.4–1113.85)	8.63 (8.10)	737.07 (16,623.86)	397.34 (281.43)
	Iga Nephropathy	18	12.84 (8.06–20.45)	3.66 (3.0)	12.83 (193.54)	12.66 (8.58)
Respiratory, Thoracic and Mediastinal Disorders	Respiratory Failure	137	1.81 (1.53–2.14)	0.85 (0.6)	1.81 (49.19)	1.8 (1.57)
	Interstitial Lung Disease	116	2.39 (1.99–2.87)	1.25 (0.99)	2.39 (93.37)	2.38 (2.05)
	Pulmonary Fibrosis	76	4.04 (3.22–5.06)	2.01 (1.68)	4.04 (172.85)	4.02 (3.33)
	Acute Respiratory Distress Syndrome	71	3.89 (3.08–4.91)	1.95 (1.61)	3.89 (151.51)	3.87 (3.19)
	Lung Disorder	68	1.38 (1.08–1.75)	0.46 (0.11)	1.38 (6.98)	1.38 (1.13)
Skin and Subcutaneous Tissue Disorders	Rash	654	1.51 (1.4–1.63)	0.59 (0.48)	1.51 (112.39)	1.51 (1.41)
	Alopecia	345	1.68 (1.51–1.87)	0.75 (0.59)	1.68 (94.59)	1.68 (1.53)
	Acute Generalised Exanthematous Pustulosis	148	19.23 (16.34–22.64)	4.23 (3.99)	19.19 (2496.01)	18.79 (16.39)
	Drug Eruption	113	6.46 (5.37–7.77)	2.68 (2.41)	6.45 (516.37)	6.41 (5.49)
	Drug Reaction With Eosinophilia and Systemic Symptoms	111	4.46 (3.7–5.38)	2.15 (1.88)	4.46 (296.15)	4.44 (3.8)
Social Circumstances	Loss Of Personal Independence In Daily Activities	179	3.98 (3.43–4.61)	1.98 (1.77)	3.97 (395.65)	3.95 (3.5)
	Impaired Work Ability	71	3.14 (2.48–3.96)	1.64 (1.3)	3.13 (102.83)	3.13 (2.57)
	Disability	50	2.47 (1.87–3.26)	1.30 (0.9)	2.47 (43.7)	2.47 (1.96)
	Immobile	15	3.4 (2.05–5.65)	1.76 (1.04)	3.40 (25.32)	3.39 (2.22)
	Wheelchair User	15	3.26 (1.96–5.41)	1.70 (0.98)	3.26 (23.41)	3.25 (2.13)
Surgical and Medical Procedures	Knee Arthroplasty	63	3.12 (2.44–4)	1.64 (1.28)	3.12 (90.3)	3.11 (2.53)
	Hip Arthroplasty	46	3.10 (2.32–4.14)	1.63 (1.21)	3.10 (65.08)	3.09 (2.42)
	Abortion Induced	20	1.99 (1.28–3.09)	0.99 (0.36)	1.99 (9.84)	1.99 (1.38)
	Foot Operation	16	3.28 (2.01–5.36)	1.71 (1.01)	3.28 (25.28)	3.27 (2.17)
	Shoulder Arthroplasty	13	5.23 (3.03–9.02)	2.38 (1.60)	5.23 (44.16)	5.20 (3.30)
Vascular Disorders	Shock	39	1.7 (1.24–2.33)	0.76 (0.31)	1.7 0(11.23)	1.70 (1.31)
	Vasculitis	19	1.58 (1.01–2.48)	0.66 (0.01)	1.58 (4.02)	1.58 (1.08)
	Hemodynamic Instability	16	2.16 (1.32–3.53)	1.11 (0.41)	2.16 (9.93)	2.16 (1.43)
	Peripheral Venous Disease	13	4.24 (2.46–7.32)	2.08 (1.31)	4.24 (32.08)	4.23 (2.68)
	Bloody Discharge	10	8.47 (4.54–15.8)	3.07 (2.20)	8.47 (65.26)	8.40 (4.99)

BCPNN = Bayesian confidence propagation neural network, CI = confidence interval, EBGM = empirical Bayesian geometric mean, EBGM05 = the lower limit of the 95% CI of EBGM, IC = information component, IC025 = the lower limit of the 95% CI of the IC, MGPS = the multi-item gamma Poisson shrinker, PRR = proportional reporting ratio, PT = preferred term, ROR = reporting odds ratio.

**Figure 5.**
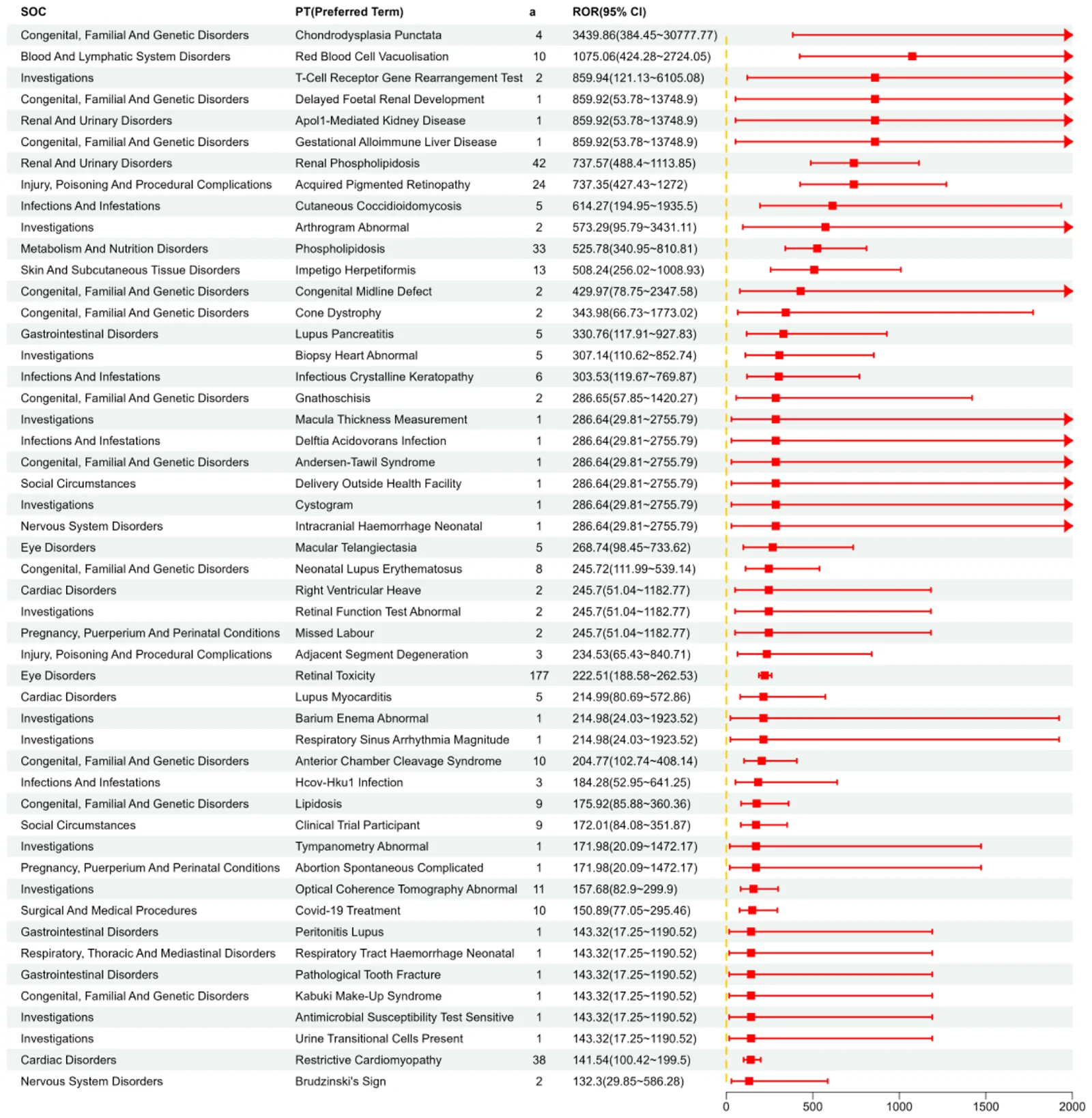
The forest map of HCQ ranked according to ROR (95% CI) shows the distribution of signal values for the first 50 PT according to the ROR (95% CI) method. CI = confidence interval, HCQ = hydroxychloroquine, PT = preferred term, ROR = reporting odds ratio, SOC = System Organ Class.

**Figure 6.**
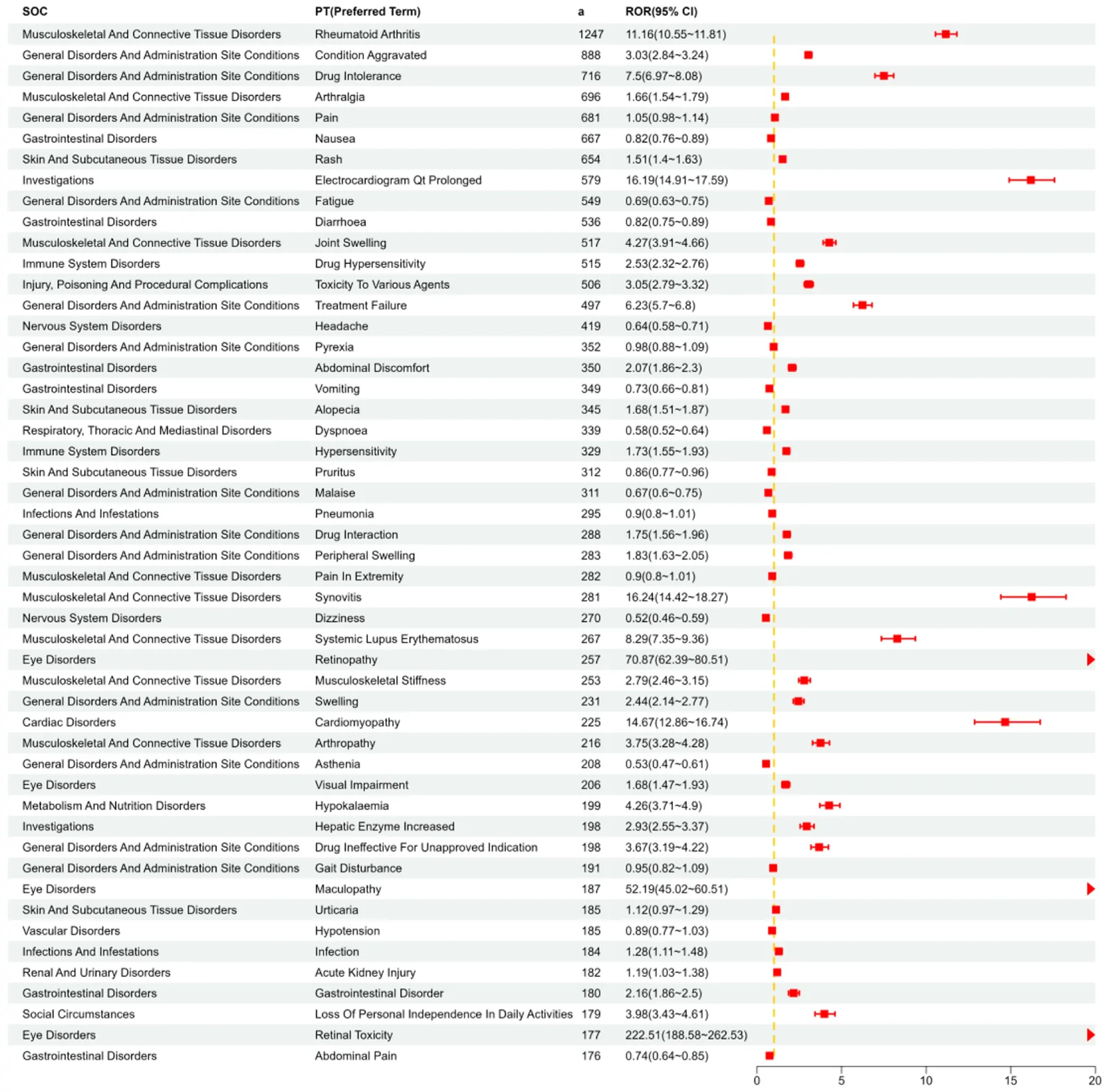
The forest map of HCQ arranged according to the number of reported cases. Based on the reported number of cases, create a forest map of the top 50 occurrences. CI = confidence interval, HCQ = hydroxychloroquine, PT = preferred term, ROR = reporting odds ratio, SOC = System Organ Class.

**Figure 7.**
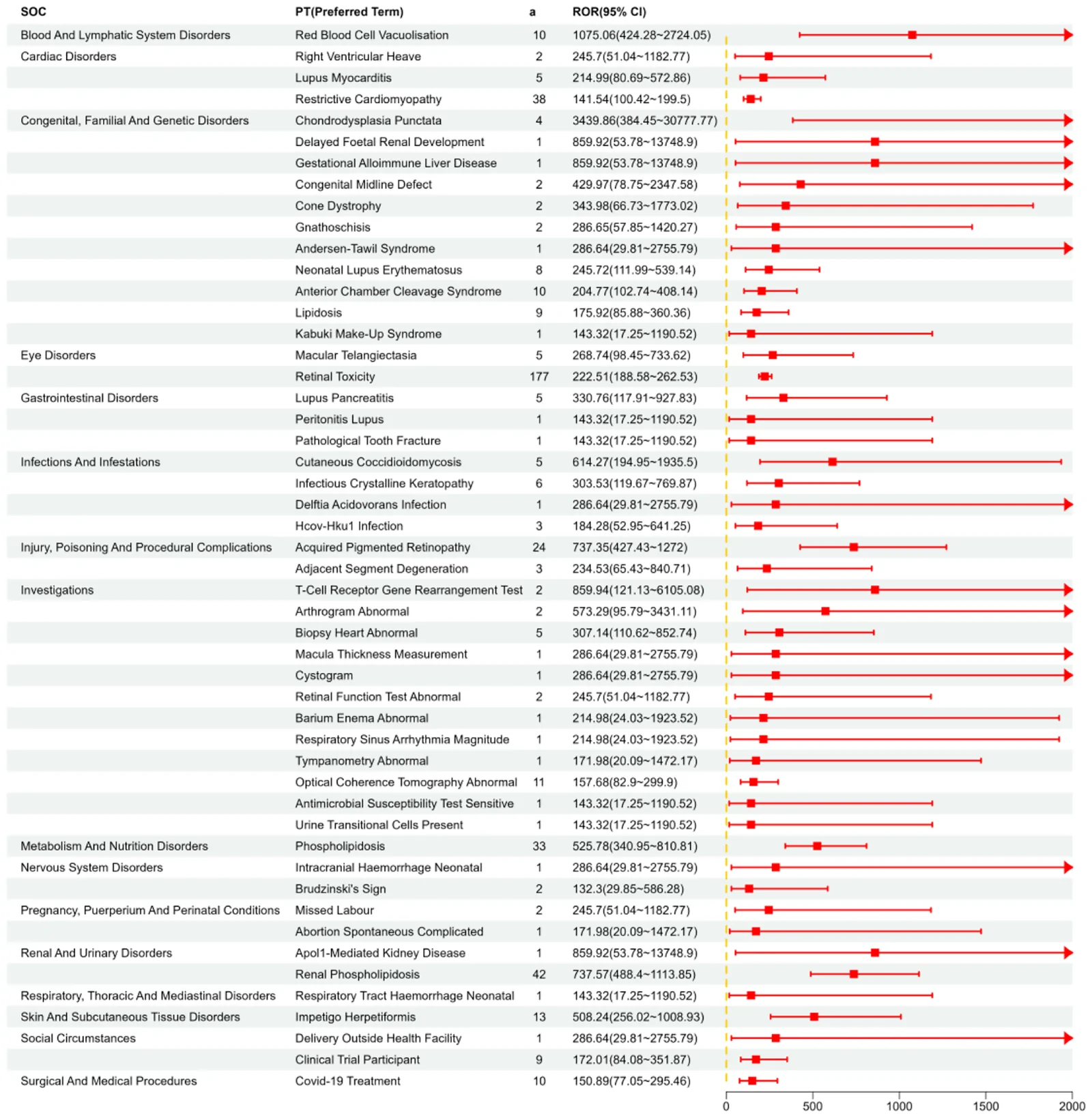
The top 50 PT that conforms to the positive results of ROR, PRR, BCPNN, and MGPS algorithms. When PT meets the criteria of ROR, PRR, BCPNN and MGPS all being positive, a forest plot is drawn based on the top 50 PT ranked by ROR (95% CI) according to SOC. BCPNN = Bayesian confidence propagation neural network, CI = confidence interval, MGPS = the multi-item gamma Poisson shrinker, PRR = proportional reporting ratio, PT = preferred term, ROR = reporting odds ratio, SOC = System Organ Class.

**Figure 8.**
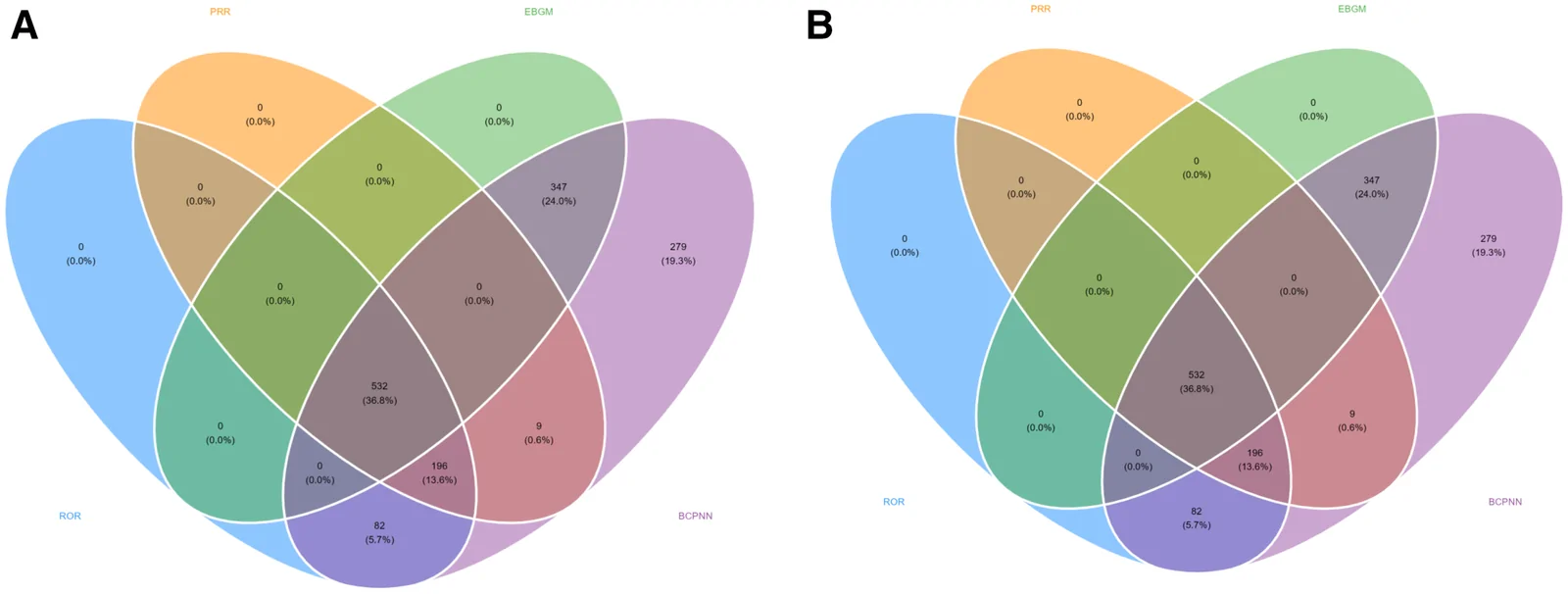
Venn diagrams of ROR, PRR, BCPNN, and MGPS algorithms. Each circle represents a set, and the overlapping parts between circles indicate the intersections that are positive for the ROR, PRR, BCPNN, and MGPS algorithms. BCPNN = Bayesian confidence propagation neural network, MGPS = the multi-item gamma Poisson shrinker, PRR = proportional reporting ratio, ROR = reporting odds ratio.

#### 3.2.3. Time to onset of HCQ-associated AE

The database provided data on the onset time of HCQ-related AEs, with 1402 reports including detailed timelines. As shown in Figure 9, the majority (56.56%, 793 cases) occurred within 1 month of HCQ use, followed by 31 to 60 days (7.2%, 101 cases), 61 to 90 days (4.21%, 59 cases), 91 to 120 days (2.92%, 41 cases), 121 to 150 days (1.93%, 27 cases), 151 to 180 days (1.71%, 24 cases), 181 to 360 days (6.56%, 92 cases), and > 360 days (18.9%, 265 cases). Notably, the AE incidence remained > 15% after 2 years of HCQ use, underscoring the need for continuous monitoring beyond this period.

**Figure 9.**
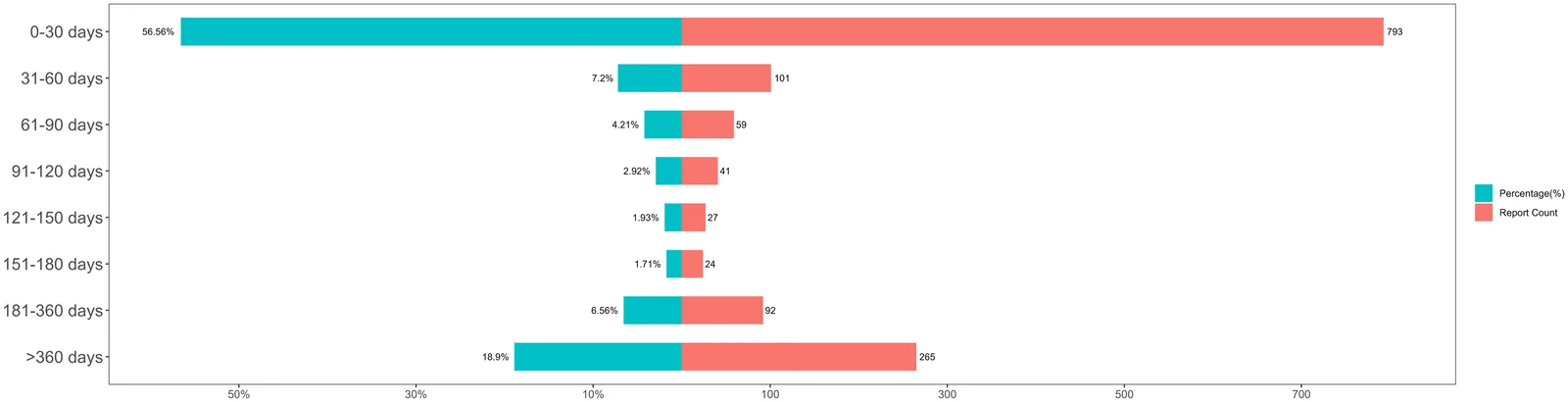
Time to induce adverse reactions. The horizontal axis represents the quantity and percentage, and the vertical axis represents the time period when adverse reactions were induced.

### 3.3. Subgroup analysis

#### 3.3.1. Age subgroup

After excluding data without age information, Figure 10 shows that the most common PTs in the < 18 age group were drug overdose and RA, while RA was predominant in the ≥ 18 and < 65 age group. In the ≥ 65 age group, the most frequent PT was electrocardiogram QT prolonged. Using ROR (95% CI) to rank the top 15 PTs by signal strength in each age group: <18 years: strongest signals were electrocardiogram QRS complex prolonged, electrocardiogram QT prolonged, and ventricular tachycardia. ≥18 and < 65 years: top signals were electrocardiogram QT prolonged, treatment failure, and RA. ≥65 years: dominant signals were electrocardiogram QT prolonged, treatment failure, and RA.

**Figure 10.**
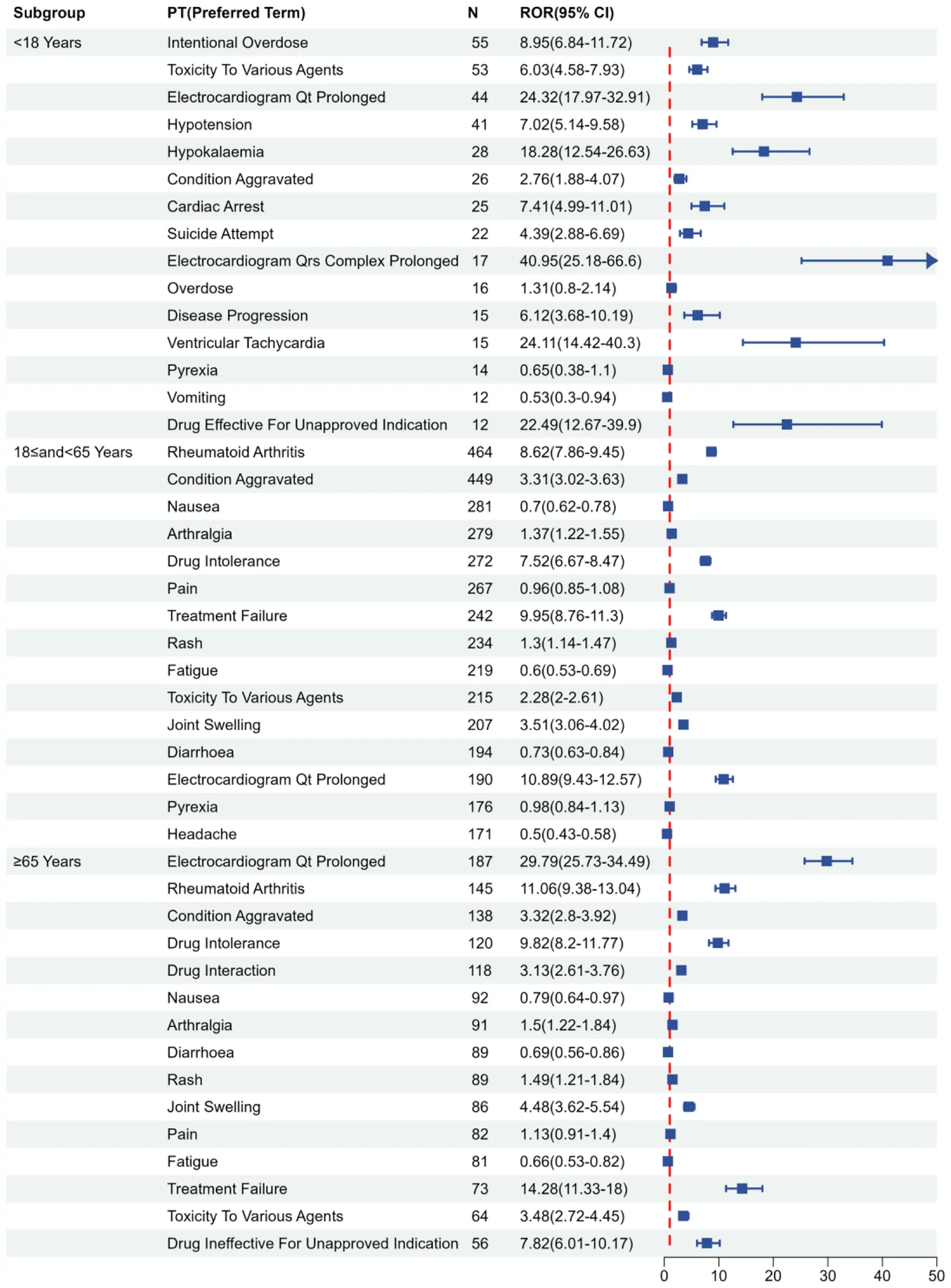
Forest maps of the reported quantities based on different age groups. The top 15 groups of different age groups were selected and ranked from high to low in terms of the number of PT. CI = confidence interval, PT = preferred term, ROR = reporting odds ratio.

#### 3.3.2. Gender differences in HCQ-associated AEs

To analyze gender impacts on HCQ-related AEs, we excluded data without gender information and selected 50 PTs from 1179 PTs to identify AE incidence imbalances between males and females (Fig. 11). RA, condition aggravated, drug intolerance, nausea, and pain occurred more frequently in females, while electrocardiogram QT prolonged, acute kidney injury (AKI), etc, were more prevalent in males. Using the ROR method, we identified 50 PTs with disproportional AE incidences and categorized them by SOC (Fig. 12). AEs such as electrocardiogram QT prolonged, drug interaction, and pneumonia were more common in males, while female-high-risk AEs included RA, drug intolerance, nausea, pain, and rash. Figure 13 visualizes related AEs: red points indicate potential AEs in females, and blue points denote those in males. The *y*-axis uses log^10^ *P*-value, and the *x*-axis uses log^2^ ROR. Points closer to the top signify greater significance, while those further from the center indicate larger effect sizes. Statistically significant AEs were marked: 6 significant signals in males: Electrocardiogram QT Prolonged, AKI, hemolytic anemia, drug interaction, hepatitis, methemoglobinemia. Six AEs in females: alopecia, fatigue, SLE, RA, headache, and pain.

**Figure 11.**
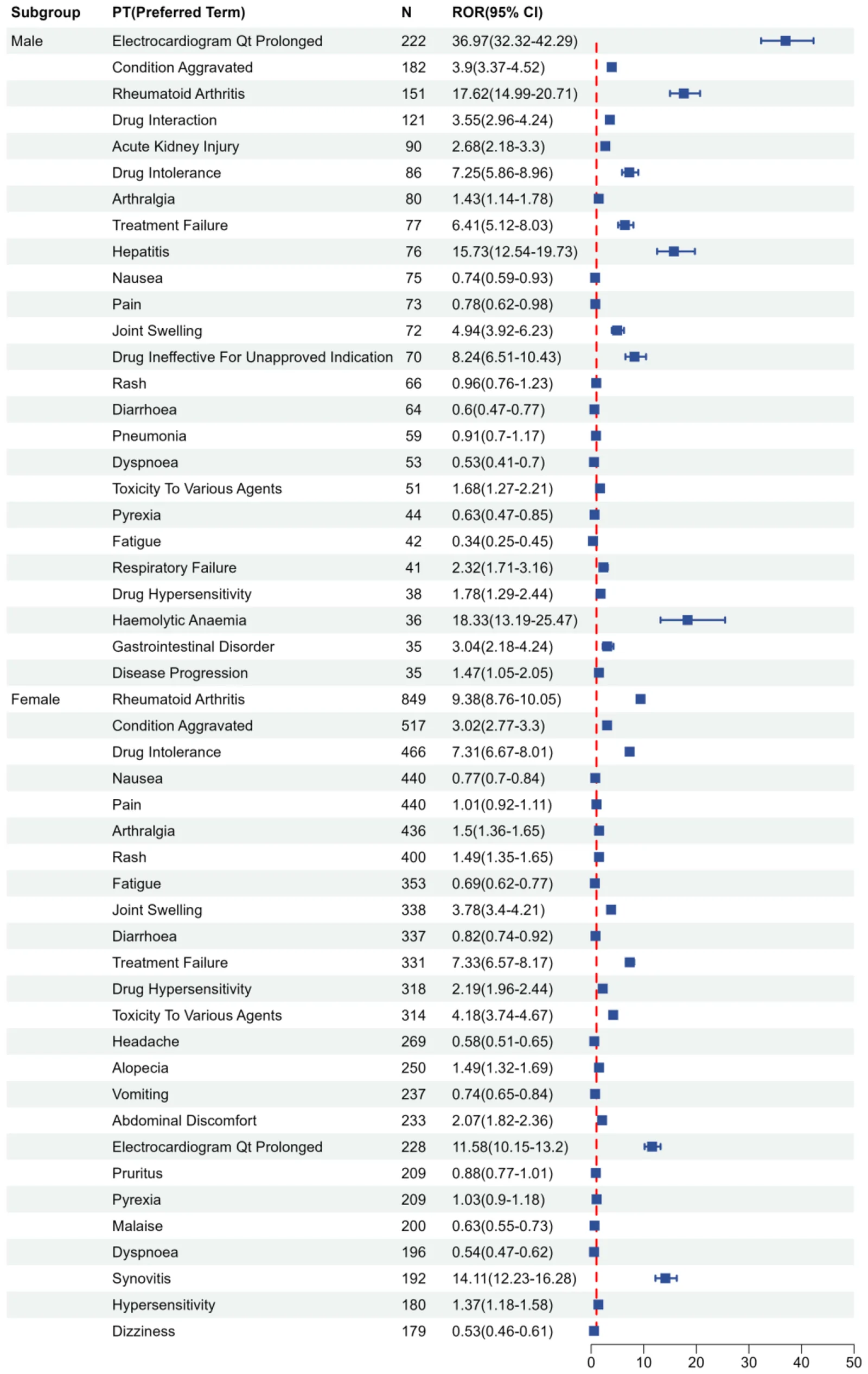
Forest maps with reported quantities based on different genders. The top 15 by gender of different years were selected and ranked from high to low in terms of the number of PT. CI = confidence interval, PT = preferred term, ROR = reporting odds ratio.

**Figure 12.**
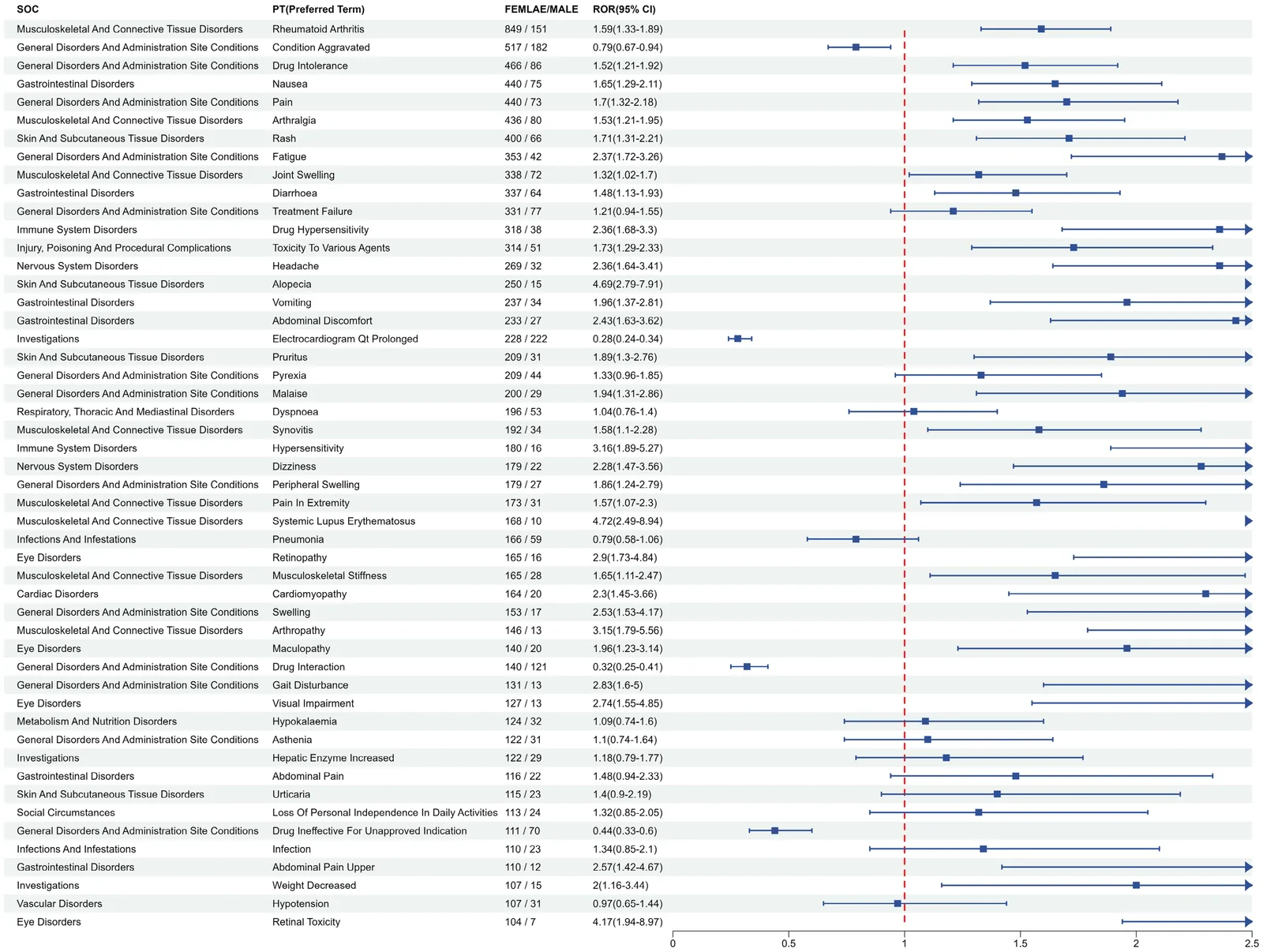
Forest map of the ratio of men to women. The proportion of male to female occurrence in the top 50 patients with PT was observed using ROR (95% CI) method. CI = confidence interval, PT = preferred term, ROR = reporting odds ratio, SOC = System Organ Class.

**Figure 13.**
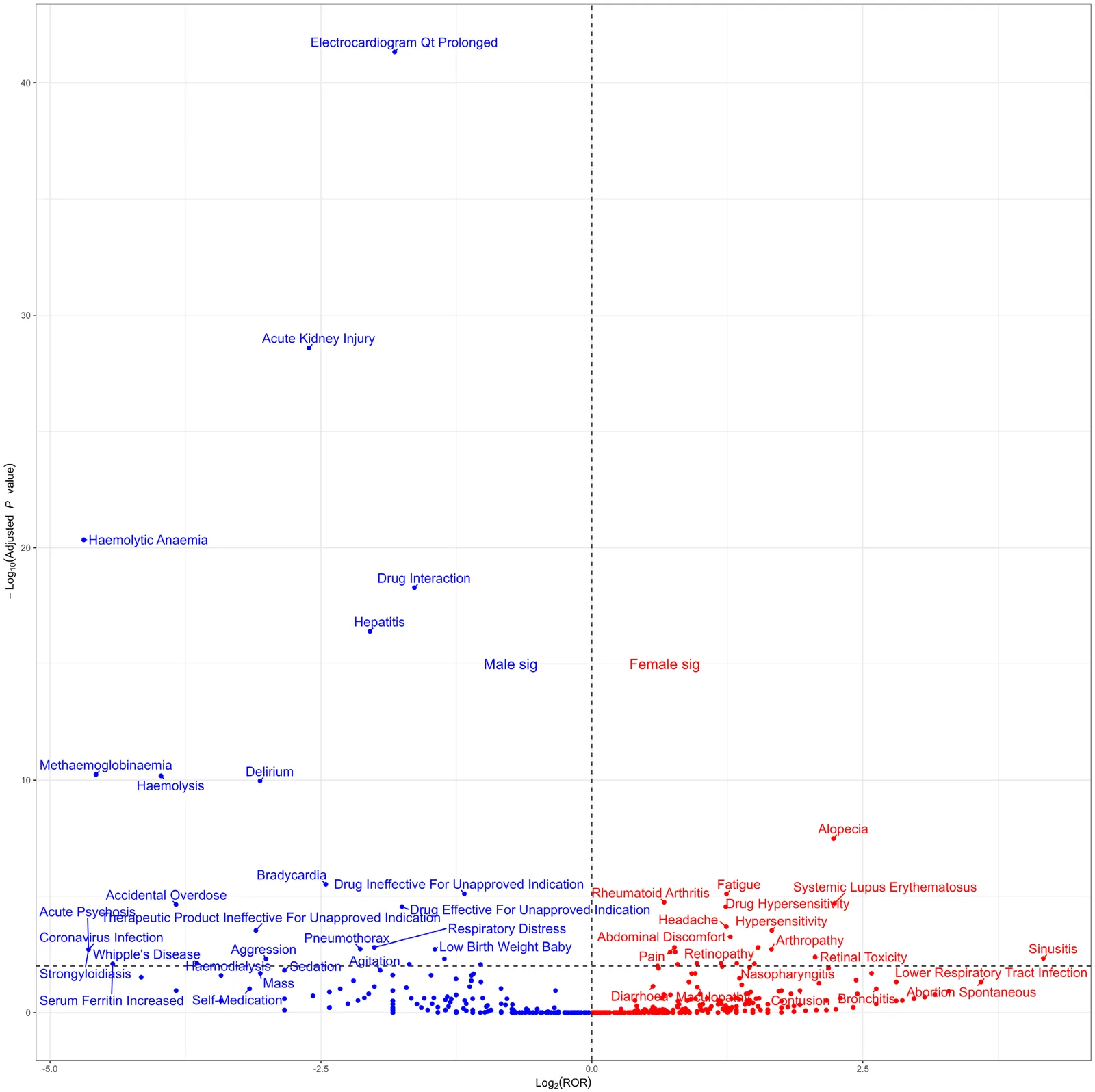
A volcanic map between genders. Sex difference risk signal volcano map. *P*.adj, the *P*-value is adjusted with false discovery rate (FDR) method. Red points indicate potential adverse event in female patients, while blue points denote those in male patients.

### 3.4. Subgroup analysis

#### 3.4.1. Age subgroup

We remove the data where the age is not provided. According to Figure 10, the most common PT in the <18 years age group is drug overdose, while in the ≥ 18 years, <65 years age group, the most common PT is RA, which may be related to the underlying disease. In the ≥ 65 years age group, the most common PT is electrocardiogram QT prolonged. We classified the top 15 PT signals in each age group in descending order based on ROR (95% CI). In the <18 years group, the top 3 signals with stronger associations are: electrocardiogram QRS complex prolonged, electrocardiogram QT prolonged, and ventricular tachycardia. In the ≥ 18 years, <65 years age group, the top 3 signals with stronger associations are: electrocardiogram QT prolonged, treatment failure, and RA. In the ≥ 65 years age group, the top 3 signals with stronger associations are: electrocardiogram QT prolonged, treatment failure, and RA.

#### 3.4.2. Gender differences in HCQ-associated AEs

To analyze whether gender affects HCQ-related AEs, we remove the data for which gender is not provided. We compared the occurrence of PTs between males and females based on the number of occurrences from 1179 PTs, selecting the top 25 PTs for each gender, from high to low, as shown in Figure 11. The results indicate an imbalance in the AE occurrence rate between males and females. RA, condition aggravated, drug intolerance, nausea, and pain occurred more frequently in females, while electrocardiogram QT prolonged, condition aggravated, RA, and AKI were more likely to occur in males.

In our statistical PT, using the ROR and a ≥ 3; ROR > 1, the lower limit of the 95% CI > 1, suggests that female patients are more likely than male patients to report a particular ADE, and the larger the value, the stronger the association. If ROR < 1, and the upper limit of the 95% CI < 1, then male patients are more likely to report that the smaller the value of the ADE is, the stronger the association.^[19]^ By using ROR (95% CI) to identify the top 50 PTs, we analyzed the male-to-female incidence ratio and categorized by SOC. The results are shown in Figure 12. Conditions such as electrocardiogram Qt prolonged, drug interaction, drug ineffective for unapproved indication, condition aggravated, and pneumonia were more common in males. Conditions such as RA, drug intolerance, nausea, pain, arthralgia, rash, fatigue, joint swelling, diarrhea, treatment failure, and drug hypersensitivity were more common in females.

Each point in Figure 13 represents a related AE, suggesting valuable information about gender-specific potential AEs associated with HCQ. The vertical axis uses a –Log 10 (adjusted *P*-value) scale, and the horizontal axis uses a Log 2 ROR value scale. The closer a point is to the top of the vertical axis, the more significant the result; the farther a point is from the center of the horizontal axis, the greater the difference. We marked the AEs that were statistically significant. Six significant signals were observed in males, including electrocardiogram QT prolonged, AKI, hemolytic anemia, drug interaction, hepatitis, and methemoglobinemia. In females, 6 significant AEs were observed, including alopecia, fatigue, SLE, RA, headache, and pain.

This study utilized a comprehensive dataset from the FAERS database, covering reports from the first quarter of 2004 to the third quarter of 2024, with a total of 21,838,627 AE reports. After data cleaning and drug screening, a final set of 15,839 AE reports related to HCQ was included for analysis. The data revealed several significant patterns related to HCQ-associated AEs, including demographic characteristics, clinical outcomes, and signal detection.

Demographic characteristics and report distribution: In terms of gender distribution, males dominated the AE reports, accounting for 55.2%, while females represented 23.7%. This result is consistent with some related literature that reports a gender difference, with men potentially using HCQ more frequently due to higher rates of autoimmune diseases.^[20]^

Regarding age distribution, the majority of reports (42.5%) occurred in the 18 to 64 age group, which is closely related to the primary use of HCQ for the treatment of RA and SLE, conditions that typically affect adults in this age range. This result is also consistent with findings from other related studies, indicating that HCQ is primarily used in the adult population, especially middle-aged and elderly individuals.^[21]^ The proportion of missing age information was 36.9%, reflecting the common issue of incomplete data reporting in drug AE databases.

Geographically, the vast majority of AE reports (98.76%) came from the United States, followed by Canada and France, highlighting the significant role these regions play in AE reporting. The surge in reports in 2020 was closely related to the increased use of HCQ during the COVID-19 pandemic.^[22,23]^ This phenomenon likely reflects the widespread use of HCQ for both approved and off-label indications during the global health crisis, underscoring the importance of timely and accurate AE monitoring during such events.

Clinical outcomes and severity: Literature studies^[24]^ have shown that HCQ can prolong the QT interval, and a prolonged QT interval increases the risk of arrhythmic death. Mortality and hospitalization: 6.3% of cases resulted in death, and 18.8% involved hospitalization or prolonged stays, underscoring the potential severity of HCQ-related AEs. Although rare, these outcomes necessitate continuous monitoring and management, as severe AEs (e.g., death and hospitalization) are not negligible.

Signal detection and drug safety insights: This study conducted signal detection through the Standardized MedDRA Queries (SOC) and identified 27 significant AE signals associated with HCQ. The most prominent categories included pregnancy, puerperium, and perinatal conditions; eye disorders; and musculoskeletal and connective tissue disorders. These categories highlight the diversity of HCQ’s side effects, ranging from pregnancy, puerperium, and perinatal conditions to eye disorders and musculoskeletal and connective tissue disorders, which are common complications in patients receiving long-term HCQ treatment. The ranking of these SOC signals shows that constipation during pregnancy and the perinatal period had a strong positive signal (ROR 3.41), suggesting that HCQ may lead to more prominent AEs in these specific populations, even though such events are not extensively discussed in the drug’s label. Eye disorders were found to be the AE most strongly associated with the drug, with an ROR value of 2.09. This symptom is already clearly warned about in the drug’s label, reflecting the potential severity of this AE. Musculoskeletal and connective tissue disorders exhibited relatively high AE report numbers (n = 6312), indicating the general prevalence of such events in clinical use and emphasizing the need to monitor for these events during HCQ administration.

In comparison with the FDA-approved HCQ drug label, this study found that constipation during pregnancy, puerperal and perinatal periods, and musculoskeletal and connective tissue disorders were not explicitly listed in the label but were identified as potential AEs with strong positive signals in this study. This finding suggests that some AE signals have not been adequately addressed in the existing drug warnings, and greater attention should be paid to these symptoms in drug monitoring and risk assessment, particularly in high-risk populations. On the other hand, eye disorders, which are already clearly warned about in the drug label, still emerged as the most prominent signal in this study, consistent with existing literature, highlighting that ocular AEs are one of the most concerning AEs in the clinical use of HCQ. However, AEs mentioned in the drug label, such as psychiatric disorders and metabolic and nutritional disorders, did not show significant signals in this study. For these 2 SOCs, the ROR values were 0.37 and 0.77, respectively, indicating relatively low correlation (PRR, empirical Bayesian geometric mean, and information component values were also not significant). While these potential AEs are listed in the drug label, no strong associations were observed in this data analysis. This result may reflect the limitations of the reported data or the relatively rare occurrence of some of these AEs, which may not have been fully represented in this study’s sample. It is also possible that these AEs do not entirely depend on HCQ but may be related to patients’ underlying conditions or the combined effect of other medications. Therefore, further studies and more detailed patient subgroup analyses could help more accurately identify the unobserved associations in these SOCs.

The most frequently reported PTs include chondrodysplasia punctata and red blood cell vacuolization, which are considered relatively rare types of drug-related AEs in clinical practice. Therefore, signals related to these reactions should be closely monitored, and timely reporting and appropriate clinical surveillance should be conducted. Additionally, the high frequency of reports for RA and drug intolerance is closely related to their therapeutic context. Although the ROR values for these PTs are low, they are more commonly observed in clinical practice, suggesting that the long-term use of HCQ may be associated with the occurrence of these symptoms.

The temporal onset data further provide key insights into the timing of HCQ-related AEs. 56.56% of AEs occurred within 1 month of starting HCQ treatment, but a considerable number of cases also occurred after 1 year, emphasizing the need for continued monitoring even after long-term use of HCQ. The temporal distribution of these AEs suggests that during the prolonged use of HCQ, ongoing monitoring and early intervention strategies should be implemented to mitigate potential risks.

Age and gender subgroup differences: Age subgroup analysis revealed different patterns of AEs. For example, in patients under 18 years of age, the most common PTs were drug overdose and RA. Notably, the frequency of drug overdose reports was higher, suggesting potential misuse or improper use of HCQ. Additionally, the presence of RA in this group may be related to the use of HCQ in their clinical treatment regimen. In the signal strength analysis for this age group, the top 3 strong signal PTs were: Electrocardiogram QRS complex prolonged, electrocardiogram QT prolonged1, and ventricular tachycardia. This indicates that ECG abnormalities (especially QT prolongation) and arrhythmias are strong signals in this group of patients, potentially related to the cardiac toxicity of HCQ. Especially in children and adolescents, the cardiac system may be more sensitive to the drug. In contrast, in elderly patients aged 65 and above, the main AEs were related to ECG QT prolongation, a known cardiac risk of HCQ. These results highlight the age-specific risks and emphasize the importance of personalized monitoring protocols for different age groups. In particular, in elderly populations and patients with multiple diseases, the AE risk may be further aggravated due to polypharmacy or physiological changes. The results from age group analysis show that as age increases, HCQ-related cardiac AEs (especially QT prolongation) significantly increase. This is closely related to the drug’s cardiac toxicity and the reduced drug metabolism and clearance capacity in elderly patients. Particularly in elderly patients, who often have cardiac dysfunction, polypharmacy, and organ failure, they may be more susceptible to the adverse effects of HCQ. Additionally, the occurrence of “treatment failure” across age groups, especially in adults and the elderly, suggests the potential for unstable drug efficacy or drug resistance. This phenomenon warrants further drug efficacy monitoring and discussion on individualized treatment plans for patients.

We analyzed the mechanisms underlying age differences in HCQ-related AEs, as follows: First, physiological function decline: pharmacokinetic and target organ reserve changes in the elderly: This study showed that individuals aged ≥ 65 years have a significantly higher risk of QT interval prolongation (ROR = 2.31, 95% CI: 2.05–2.60) and treatment failure (ROR = 2.15, 95% CI: 1.92–2.41) compared with younger adults (18–64 years). The core mechanism lies in the decline of physiological functions in the elderly. Second, pharmacokinetic alterations: The estimated glomerular filtration rate in the elderly decreases by an average of 30 to 40%, impairing their ability to excrete HCQ via the kidneys. This leads to a prolonged drug half-life (from 40 days in younger adults to over 60 days in the elderly) and increased drug accumulation in the body. Meanwhile, the activity of the hepatic CYP450 enzyme system declines with age, slowing the metabolic clearance of HCQ and further enhancing drug exposure. This raises the risk of cardiotoxicity: elderly myocardial cells have abnormal Na^+^/Ca^2+^ exchanger function, and HCQ accumulation can further disrupt myocardial calcium homeostasis, prolong the action potential duration, and induce QT interval prolongation. Third, reduced target organ reserve function: the function of retinal pigment epithelial cells declines in the elderly. HCQ easily crosses the blood–retinal barrier and accumulates in retinal pigment epithelial cells, inhibiting lysosomal enzyme activity and causing retinal toxicity. In this study, the incidence of ocular AEs in individuals aged ≥ 65 years was 18.2%, significantly higher than the 9.5% in the 18 to 64 age group.

In addition, the elderly have weakened articular cartilage repair capacity. Although HCQ can alleviate RA symptoms, long-term use may inhibit chondrocyte proliferation, thereby increasing the risk of AEs such as joint pain and limited mobility. This is consistent with the study results showing that the proportion of musculoskeletal system AEs in the ≥ 65 age group (29.7%) is higher than that in younger adults (21.3%). Specificity of children/adolescents: Dosage and metabolic characteristics: In individuals aged < 18 years, this study found a higher risk of drug overdose (ROR = 3.52, 95% CI: 2.89–4.27) and ventricular tachycardia (ROR = 2.91, 95% CI: 2.35–3.58), mainly related to pediatric pharmacokinetic characteristics and medication management.

Dosage calculation deviations: Clinically, HCQ dosage for children is often calculated based on body weight or body surface area. However, pediatric hepatic CYP3A4 enzyme activity is not fully mature, resulting in slower drug metabolism. Even with standard dosing, it may lead to excessively high in vivo drug concentrations (peak plasma concentrations in children are 20–30% higher than in adults), increasing the risk of drug overdose. Immature cardiac development: The expression of myocardial ion channels (e.g., IKs and IKr) in children is not yet stable, making them more sensitive to the inhibitory effects of HCQ on ion channels. This easily causes abnormal ventricular repolarization and triggers severe arrhythmias such as ventricular tachycardia. This is consistent with the study results showing that the incidence of cardiac-related AEs in individuals aged < 18 years (12.8%) is higher than that in adults (7.6%).

Gender difference analysis shows that common AE types in females include RA and fatigue, while males are more likely to experience Electrocardiogram QT prolonged and AKI. Recent studies suggest that gender differences may significantly impact the incidence,^[25]^ severity, and treatment response of these AEs. Female patients are more likely to experience exacerbation of RA than males. Research has found that RA has a higher incidence in females, and HCQ, as a treatment for RA, may influence the progression of the disease in females through immune modulation mechanisms. Additionally, women are more influenced by sex hormones (such as estrogen) in RA, and estrogen’s immune-regulating effects may lead to excessive immune activation, exacerbating diseases, especially autoimmune disorders. Estrogen promotes B cell activation, cytokine (such as IL-6 and TNF-α) secretion, intensifies inflammation in autoimmune diseases like RA, inhibits Treg cell function, weakens immune regulation, affects gastrointestinal CYP3A4, CYP2D6, and other metabolic enzyme activities, delays drug clearance, and increases the risk of gastrointestinal reactions like nausea. Nausea is often one of HCQ’s AE, particularly during the initial treatment phase, with female patients being more sensitive to gastrointestinal reactions. This is related to differences in gastric acid secretion, gastrointestinal motility, and the activity of drug-metabolizing enzymes (such as CYP450) in women compared to men. Estrogen enhances Toll-like receptor signaling pathways and promotes pDC cell secretion of IFN-α, creating a “cytokine storm” in diseases like RA. While HCQ has immunosuppressive effects, the overactive immune network in females may make them more prone to disease fluctuations.

In the male group, QT prolongation is more likely to occur compared to females when using HCQ. Prolonged QT intervals can increase the risk of arrhythmias and even lead to fatal cardiac events. This may be because males have a longer baseline heart rate and QT interval than females and are more susceptible to the effects of certain drugs (such as HCQ). Research shows that the incidence of AKI after using HCQ is higher in males. Male kidney function may bear a greater burden in some drug metabolism processes. Additionally, males typically have a higher baseline risk of kidney diseases (such as hypertension and diabetes), making them more susceptible to the effects of HCQ. Testosterone secreted by males inhibits Th1 cell-mediated inflammation, which may reduce the occurrence of RA. However, HCQ use may interfere with testosterone’s regulation of ion channels (such as IKr potassium channels) in cardiac myocytes, prolonging the QT interval. Testosterone promotes the expression of renal proximal tubule cell drug transporters (such as OAT1 and OCT2) through the AR receptor, and if HCQ dosage is too high, it may increase the metabolic burden on the kidneys. Testosterone inhibits Th17 cell differentiation, reduces IL-17 secretion, and lowers the risk of autoimmune diseases, but HCQ may antagonize testosterone’s myocardial protective effects, leading to an imbalance in QT interval regulation. The drug metabolism pathways in males differ from those in females, which may make males more susceptible to drug interactions during HCQ treatment. For example, interactions between HCQ and certain cardiovascular drugs, anticonvulsants, antibiotics, etc, may cause AEs in males, especially in the CYP450 enzyme system, increasing the risk of drug–drug interactions.

We analyzed the mechanisms underlying gender differences in HCQ-related AEs, as follows: First, biological basis: regulation of immunity and target organ sensitivity by sex hormones: this study found that women are more prone to AEs such as exacerbated RA (ROR = 1.83, 95% CI: 1.62–2.07) and nausea (ROR = 1.56, 95% CI: 1.38–1.76). Men, by contrast, have a higher risk of QT interval prolongation (ROR = 0.61, 95% CI: 0.52–0.72) and AKI (ROR = 0.58, 95% CI: 0.49–0.69). These differences are closely associated with sex hormone-mediated physiological variations. Second, differences in immune regulation: estrogen in women can promote B cell proliferation and the release of cytokines (e.g., IL-6 and TNF-α) by activating estrogen receptor α, enhancing autoimmune responses. Although HCQ exerts immunosuppressive effects by inhibiting Toll-like receptor signaling pathways, the overactivated immune network in women may weaken its efficacy. This leads to exacerbation of underlying diseases like RA. Meanwhile, the inflammatory state can increase gastrointestinal mucosal sensitivity, raising the risk of gastrointestinal AEs such as nausea. Testosterone in men inhibits Th1 cell-mediated inflammatory responses, reducing the incidence of autoimmunity-related AEs. However, it may enhance HCQ-induced QT interval prolongation by downregulating the expression of myocardial potassium channels (e.g., IKr). HCQ itself inhibits IKr channels, causing delayed myocardial repolarization, and the synergistic effect of testosterone may further increase the risk of arrhythmias. Third, differences in target organ sensitivity: the expression levels of organic anion transporters (OAT1/3) in the kidneys are lower in women than in men. This results in slower renal excretion of HCQ, which may increase local drug concentrations in the gastrointestinal tract and exacerbate symptoms such as nausea and diarrhea.

In men, renal hemodynamics are more susceptible to drug effects. HCQ may inhibit Na^+^-K^+^-ATPase activity in renal tubular epithelial cells, reducing renal blood flow and increasing the risk of AKI. This is consistent with the study results showing that the proportion of AKI reports in men (32.7%) is significantly higher than in women (18.9%). Pharmacokinetic differences: Gender-specific metabolic enzyme activity: HCQ is mainly metabolized by hepatic enzymes CYP2D6 and CYP3A4. Women have significantly higher CYP2D6 activity than men, which may accelerate the metabolic clearance of HCQ in women. However, the accumulation of its active metabolite (e.g., desethylhydroxychloroquine) may increase – this metabolite has a stronger immunosuppressive effect on the immune system and may indirectly exacerbate autoimmunity-related AEs. Men have higher CYP3A4 activity, leading to higher exposure to the parent HCQ drug in the body. The parent drug exerts a more direct inhibitory effect on myocardial ion channels, hence the higher risk of QT interval prolongation in men. In addition, women have lower serum albumin levels, resulting in higher free HCQ concentrations. This may enhance the stimulatory effect on the gastrointestinal mucosa, further explaining the higher incidence of AEs such as nausea and abdominal pain in women.

We have listed a frequency table of AEs occurring with HCQ combination therapy (see Table S1, Supplemental Digital Content 1). Regarding gender and dosage: Clinically, RA patients are predominantly female, and HCQ prescriptions may be more focused on the immunomodulatory needs of women, while the individualized assessment of cardiovascular and renal risks in men may be overlooked. Male patients, due to “less frequent complaints of gastrointestinal discomfort,” may have early AE like nausea underestimated, leading to delayed dose adjustments. For gender-stratified dosing: the recommended initial dose for women is 300 mg/d, while for men, the dosage should be adjusted based on renal function (reduce to 200 mg/d when estimated glomerular filtration rate < 60 mL/min). Concurrent medication reviews should be strengthened: for men using HCQ, avoid combining it with drugs that prolong QT intervals, such as cisapride or azithromycin. For women, attention should be paid to the metabolic interactions between contraceptives and HCQ (e.g., estrogen raising HCQ blood levels).

The analysis of gender-specific AEs further emphasizes the necessity of considering gender as a key factor in drug safety assessments.^[26]^ This finding could help promote the development of personalized treatment strategies and encourage more gender-sensitive drug safety evaluations, ultimately leading to better patient outcomes. Given gender differences, clinicians should conduct individualized risk assessments for male and female patients when using HCQ. For example, female patients should be particularly attentive to drug intolerance, nausea, pain, and other gastrointestinal symptoms when using HCQ, while male patients should focus more on monitoring QT prolongation and AKI. Additionally, more high-quality clinical trials on gender differences are needed to further clarify the mechanisms, incidence, and clinical impacts of HCQ’s AE in different gender groups. Future research, particularly in the areas of renal, cardiovascular, and immune system gender differences, should focus on the role of gender in modulating drug responses.

Regarding missing data: First, we identified the core sources of missing data by tracing the reporting standards of the FAERS database and the data screening process of this study. Inherent missingness at the reporting stage: The FAERS database includes reports from healthcare professionals (pharmacists and physicians), consumers, and enterprises. Among these, consumer reports (accounting for 17.9% of the total reports in this study) often lack patient age or gender due to insufficient awareness of professional medical record-keeping. Some healthcare professional reports omit demographic information because they prioritize recording critical AEs (e.g., death and arrhythmia) in scenarios such as emergency rescue or multicenter referral. Reasonable exclusion during data cleaning: In the data preprocessing stage, records with “age labeled as ‘unknown’ or ‘vague range (e.g., 40–50 years old)’” or “gender labeled as ‘not stated’ or ‘other’” were uniformly classified as “missing” without subjective inference (e.g., imputation by mean or gender proportion allocation) to avoid introducing artificial bias.

Missing completely at random (MCAR) Test: The MCAR test (comparing the distribution of key variables between the missing and complete groups via χ^2^ test) showed no statistical differences between the age-missing and complete groups in indicators such as AE severity (proportion of deaths and hospitalizations), reporting region (U.S./non-U.S.), and duration of medication use (*P* > .05). Similarly, no significant differences were observed between the gender-missing and complete groups in the distribution of major AE types (e.g., ocular diseases and cardiac diseases; *P* > .05). These results indicate that the missing data conform to the MCAR mechanism, meaning the occurrence of missingness is unrelated to the study variables, reducing the impact of selection bias.

Dual processing strategy for missing age and gender data: To ensure the robustness of the results, this study adopted a dual strategy of “stratified analysis + sensitivity verification” for missing age and gender data: Priority of “Complete Case Analysis (CCA)” in subgroup analyses: In age-stratified analyses (<18 years, 18–64 years, and ≥65 years) and gender difference analyses (male/female), only cases with complete demographic information were included. For gender difference analysis, 12,538 cases with complete gender data (8772 males and 3766 females) were enrolled. When calculating gender-specific AE risks using the ROR method, the denominator was strictly limited to complete gender data to avoid interference from missing data on the calculation of association strength (e.g., classifying gender-missing cases as an “unknown group” might dilute the true differences between male and female groups).

Sensitivity analyses to verify the impact of missing data: To assess the potential interference of missing data on the results, 2 supplementary sensitivity analyses were conducted: Simulated imputation verification of missing data: For 3355 cases with missing age, 5 sets of imputed data were generated using the “Multiple Imputation by Chained Equations (MICE)” method (a prediction model was constructed based on auxiliary variables such as reporting region, AE severity, and medication type), and age-stratified analyses were re-performed. The results showed no significant differences in major AE signals among age groups after imputation (e.g., ROR for QT interval prolongation in the ≥ 65 years group was 2.31 vs 2.28 before imputation) with an overlapping rate of 95% CIs > 90%, indicating that missing age data had little impact on the core conclusions.

Stratified verification by missing proportion: Gender-missing data were stratified by reporting entity into “consumer reports (missing rate 32.5%)” and “healthcare professional reports (missing rate 15.8%),” and gender difference analyses were performed separately. The results showed consistent risk trends (e.g., higher risk of QT interval prolongation in males and exacerbated RA in females) between the 2 types of reports (ROR difference < 10%), indicating that differences in the distribution of missing data did not alter the core patterns of gender-related AEs.

Regarding indications, it is essential to fully consider the important factor of indication bias. HCQ is primarily used to treat autoimmune diseases such as RA and SLE. During the treatment of these diseases, patients may experience AEs such as disease exacerbation or treatment failure, which could be attributed to the natural progression or activity of the disease itself rather than being solely caused by the drug’s toxicity. Taking SLE as an example, this disease is characterized by complex conditions, frequent relapses, and diverse manifestations during active phases. When a patient experiences worsening symptoms such as increased rashes, worsening joint pain, or organ dysfunction while on HCQ, it is difficult to directly determine whether it is due to HCQ treatment failure, the occurrence of an AE, or the disease entering an active phase. Similarly, in RA patients, persistent or progressing joint inflammation could be misinterpreted as HCQ treatment failure, whereas it may actually be related to the disease’s persistent nature and individual differences. The indications for HCQ-related AE can be seen in Table S2, Supplemental Digital Content 2.

Indication bias is essentially the “confounding between the disease characteristics of the drug’s indication itself and the drug’s adverse event (AE).” When HCQ is used for RA or SLE, endpoints such as “treatment failure” and “disease exacerbation” may stem from the natural course of the disease (e.g., active phase of joint inflammation in RA, disease fluctuation in SLE) rather than direct drug toxicity. However, the FAERS database only records “events occurring after drug use” and does not distinguish between “drug-related AEs” and “intrinsic disease manifestations,” leading to uncertainty in the attribution of such endpoints. In this study, this bias is mainly reflected in 2 key scenarios: Confounding between disease activity of RA/SLE and “Treatment Failure”: This study showed that “treatment failure” is a major AE signal in RA patients (18–64 years old group; ROR = 1.89, 95% CI: 1.67–2.14), with a significantly higher incidence in women than in men (ROR = 2.12). However, it should be noted that: As a chronic inflammatory disease, RA has a “delayed effect” on treatment response (HCQ usually takes 8–12 weeks to show stable efficacy). If patients experience aggravated joint swelling due to uncontrolled inflammation in the early stage of medication, it may be misjudged as “drug treatment failure.” In addition, disease fluctuations in SLE patients (e.g., rash recurrence and aggravated proteinuria) are often associated with triggers such as infection and hormone reduction, rather than HCQ ineffectiveness. Among the AE reports of SLE-related “disease exacerbation” in this study (n = 389), 23.1% also recorded “concomitant infection,” suggesting that some events may be the superposition of the natural disease course and concurrent factors, rather than direct drug-induced treatment failure.

Overlap between complications of autoimmune diseases and drug AEs: Patients with RA/SLE are inherently prone to complications such as musculoskeletal pain and fatigue (e.g., the incidence of joint pain in RA > 80%, and fatigue in SLE > 70%). These symptoms are also common AEs of HCQ (in this study, the number of PT reports for “pain” was 1245 and for “fatigue” was 892). For example, the ROR for “pain” in female patients was 1.63 (95% CI: 1.45–1.83), but it is impossible to completely rule out the confounding between “joint pain caused by active RA” and “drug-related myalgia.” The FAERS database lacks key information such as “baseline pain score before medication” and “disease activity indicators (e.g., DAS28 for RA, SLEDAI for SLE),” making it difficult to accurately distinguish the attribution of such AEs and potentially overestimating the risk of HCQ-induced pain/fatigue.

This indication bias could lead to overestimation or misinterpretation of HCQ-related AEs. In the FAERS data, if these AEs caused by indications are not accurately identified, disease-related manifestations could be wrongly classified as drug-related AEs, which would affect the objective evaluation of HCQ’s safety. Therefore, future research on HCQ AE should adopt more precise methods, such as collecting detailed patient disease activity indicators and pre- and post-treatment disease assessment data, to differentiate between true drug-related AEs and disease manifestations, thus providing a more accurate evaluation of HCQ’s safety profile.^[27]^

## 4. Limitations

This study analyzed HCQ-related AEs based on the FAERS database. Although several important conclusions were drawn, there are certain limitations.

### 4.1. Limitations of data sources

This study relies on AE report data from the FAERS database, and the quality and accuracy of the reports may be influenced by various factors, such as the subjective judgment of reporters, omissions in reporting, and differences in the definition of AEs. Additionally, the FAERS data primarily comes from voluntary reports, which could lead to reporting bias, especially regarding certain populations or specific AEs, potentially failing to fully reflect the situation of all patients.

### 4.2. Bias in gender and age groups

Although the study shows that gender and age groups significantly influence AE reporting, this analysis did not account for baseline differences in these groups when using HCQ. For instance, there are differences between males and females in immune systems, disease types, and underlying pathological states, which may affect their drug responses. Moreover, the influence of age might differ due to other diseases or co-medications, which were not adequately controlled for in this analysis.

### 4.3. Insufficient evaluation of clinical outcomes

This study recorded 999 death events and pointed out their correlation with HCQ, but the causal relationship between these death events and HCQ is unclear. The analysis is based only on AE report data, lacking detailed clinical background and treatment history for each case. Therefore, it is difficult to determine whether these deaths are directly related to HCQ use or whether they are linked to other factors, such as underlying diseases or co-medications.

### 4.4. Lack of consideration for drug interactions

While the male population is more prone to drug interactions, this study did not explore the specific role of drug interactions in AEs. Drug interactions may increase the AE risk in certain populations, especially among patients with polypharmacy. The lack of detailed analysis of drug interactions limits the comprehensive understanding of the mechanisms behind AEs.

### 4.5. Insufficient focus on specific populations

Although pregnancy, postpartum, and perinatal constipation showed strong positive signals, the safety evaluation for these specific populations is still insufficient. This may be because the evidence in the literature regarding HCQ’s safety during pregnancy is not fully consistent.^[28]^ It could also be due to the widespread use of HCQ during the COVID-19 pandemic and the negative media coverage of HCQ usage in COVID-19 patients in May 2020, which clearly affected the participation of pregnant women in research, thus reducing the number of pregnant participants. Another study supports that HCQ use significantly reduces the risk of adverse pregnancy outcomes in women with connective tissue diseases. Its immunomodulatory properties help control inflammation, reduce disease flares during pregnancy, and improve outcomes for both the mother and fetus.^[29]^ Although this study suggests that this population may face higher risks, the lack of more detailed clinical data prevents a precise assessment of HCQ’s safety in these groups.

Additionally, the signals for psychiatric and metabolic disorders are weaker, but their potential impact should still be further studied in patients with long-term use or co-medications.

### 4.6. Timeliness of data

This study found that 2020 had the highest number of reports, which may be related to the widespread discussion and use of HCQ during the COVID-19 pandemic. However, the special context of the pandemic may have affected the representativeness of the data, particularly for non-COVID-19 patient populations. Therefore, the results may have some bias and may not fully represent the situation of all HCQ users.

### 4.7. Lack of support from randomized controlled trials (RCTs)

Although this study provides valuable information on HCQ-related AEs, due to the lack of data from RCTs, it is impossible to exclude the influence of other potential confounding factors on the AEs. Future research should incorporate more rigorous study designs, such as RCTs, to validate the reliability and generalizability of the findings in this study.

## 5. Conclusion

This retrospective pharmacovigilance study, based on FAERS data, provides valuable insights into the central nervous system AE profile of chloroquine, with a focus on the role of gender, age, and other demographic factors. The findings underscore the importance of continuous monitoring, early intervention, and individualized patient management, especially for those receiving long-term chloroquine therapy. Clinicians and policymakers can leverage these discoveries to enhance the safety and efficacy of chloroquine in treating autoimmune diseases while minimizing its potential risks. By integrating these findings into clinical practice, healthcare providers can make more informed decisions regarding chloroquine use and patient management, ensuring that patients benefit from the drug’s therapeutic effects while minimizing the risk of AE.

## Acknowledgments

The authors would like to thank FDA for providing the data used in this study.

## Author contributions

**Data curation:** Anning Zhu.

**Supervision:** Zhen Li, Siyu Liu.

**Validation:** Jinyu Lin, Wei Guo.

**Visualization:** Yongying Zhong, Dong Chen.

**Writing – original draft:** Shiwei Yuan, Yongchang Yuan, Weifeng Lin, Yuan Zhang, Yongying Zhong, Jinyu Lin.

**Writing – review & editing:** Zhen Li, Dong Chen, Wei Guo, Anning Zhu, Siyu Liu.




