# Supplementary material for: Adverse drug events associated with hydroxychloroquine: A real-world pharmacovigilance study from 2004 to 2024 using the FAERS database
Source: Medicine (Baltimore). 2026 Oct 30;105(44):e51285. doi: 10.1097/MD.0000000000051285 (PMC13727192; doi:10.1097/MD.0000000000051285)
Supplement: Supplementary file 1 [file medi-105-e51285-s001.docx]

| Drug | N |
| --- | --- |
| Methotrexate | 1795 |
| Prednisone | 1471 |
| Enbrel | 1409 |
| Hydroxychloroquine Sulfate | 1370 |
| Humira | 1208 |
| Azithromycin Anhydrous | 1189 |
| Methotrexate | 1104 |
| Arava | 1032 |
| Sulfasalazine | 910 |
| Prednisone | 871 |
| Xeljanz | 807 |
| Actemra | 777 |
| Lopinavir Ritonavir | 768 |
| Hydroxychloroquine Sulfate | 756 |
| Orencia | 753 |
| Leflunomide | 646 |
| Remicade | 585 |
| Prednisolone | 560 |
| Methylprednisolone | 521 |
| Tocilizumab | 513 |
| Methotrexate Sodium | 478 |
| Ceftriaxone | 424 |
| Sulfasalazine | 393 |
| Prednisolone | 390 |
| Rituxan | 382 |
| Folic Acid | 380 |
| Simponi | 379 |
| Mycophenolate Mofetil | 361 |
| Hydroxychloroquine Sulfate Authorized Generic Plaquenil | 360 |
| Azathioprine | 356 |
| Cimzia | 352 |

Supplementary Table 1 .The frequency of adverse reactions when combined with HCQ（Top30）
