# Supplementary material for: Adverse drug events associated with hydroxychloroquine: A real-world pharmacovigilance study from 2004 to 2024 using the FAERS database
Source: Medicine (Baltimore). 2026 Oct 30;105(44):e51285. doi: 10.1097/MD.0000000000051285 (PMC13727192; doi:10.1097/MD.0000000000051285)
Supplement: Supplementary file 2 [file medi-105-e51285-s002.docx]

Supplementary Table 2. The top 30 indications for adverse reactions related to HCQ

| INDI_PT | N |
| --- | --- |
| Rheumatoid Arthritis | 3930 |
| Covid-19 | 3130 |
| Systemic Lupus Erythematosus | 2412 |
| Covid-19 Pneumonia | 695 |
| Immunosuppressant Drug Therapy | 499 |
| Prophylaxis | 324 |
| Sjogren'S Syndrome | 289 |
| Hypertension | 247 |
| Coronavirus Infection | 195 |
| Anticoagulant Therapy | 191 |
| Arthritis | 191 |
| Cutaneous Lupus Erythematosus | 144 |
| Lupus Nephritis | 139 |
| Dermatomyositis | 135 |
| Psoriatic Arthropathy | 130 |
| Pneumonia | 127 |
| Antibiotic Therapy | 125 |
| Pain | 115 |
| Antiphospholipid Syndrome | 108 |
| Thrombosis Prophylaxis | 102 |
